# Stability of potato glycoalkaloids under heating conditions – Reactions with fatty acids

**DOI:** 10.1016/j.crfs.2026.101438

**Published:** 2026-05-15

**Authors:** Keven Mittau, Christina Meyers, Harshadrai M. Rawel, Inga Smit, Marcus Schmidt, Sascha Rohn

**Affiliations:** a Technische Universität Berlin, Institute of Food Technology and Food Chemistry, Kaiserin-Augusta-Allee 14, Berlin, 10553, Germany; b Max Rubner-Institut, Federal Research Institute of Nutrition and Food, Department of Safety and Quality of Cereals, Schützenberg 12, Detmold, 32756, Germany; c University of Potsdam, Institute of Nutritional Science, Arthur-Scheunert-Allee 114-116, Nuthetal, 14558, Germany; d Eberswalde University for Sustainable Development (HNEE), Schickler Straße 5, Eberswalde, 16225, Germany

**Keywords:** Glycoalkaloid, Fatty acid, Esterification, Oxidation, Potato, Processing

## Abstract

Glycoalkaloids (GA) are endogenous metabolites of the plant family *Solanaceae* and considered as contaminants. However, their stability and reactivity during high-temperature food processing such as frying and deep-frying of potatoes has not been comprehensively studied. Consequently, the aim of this study was to characterize the reactivity of GA in heat-induced reactions by investigating the formation of novel reaction products. Based on the assumption that GA may be present in cell membranes and may react with lipids and amphiphilic compounds, in conjunction with the results of preliminary screening measurements for reaction products, this work focused on reactions with (endogenous) lipids. For this purpose, pure GA were incubated with fatty acids (palmitic, stearic, oleic, linoleic and α-linolenic acid) at temperatures commonly applied during deep-frying (180 °C). Reaction products formed were characterized by multi-stage high-resolution mass spectrometry and liquid chromatography-coupled tandem mass spectrometry. Two novel reaction pathways were identified and described – esterification of GA with fatty acids via hydroxyl groups of the sugar moiety and oxidation of the steroidal alkaloid moiety. Additionally, the formation of these products was verified in processed potato matrices, without the addition of frying oil, which indicates that the ester formation originates from the tuber's endogenous fatty acids. Consequently, the findings presented herein contribute to the understanding of the chemical fate of GA during thermal processing of potato products. However, the total amounts formed must still be determined, and a toxicological study must then be conducted to assess the extent to which the reaction products affect on human nutrition.

## Highlights

• Fatty acids are main reactants in the heat-induced conversion of α-glycoalkaloids.
• GA react faster with polyunsaturated than with saturated fatty acids.
• Esters and oxidation products were identified as novel GA conjugates in potato products.
• Residual potato peels might lead to a pronounced formation of reactions products.
• The formation of a large number of related compounds in potato products is expected.

## Graphical abstract

**Figure:**
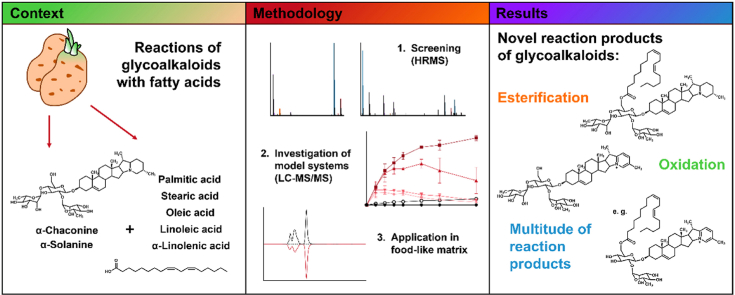

## 1 Introduction

**Fig. 1:**
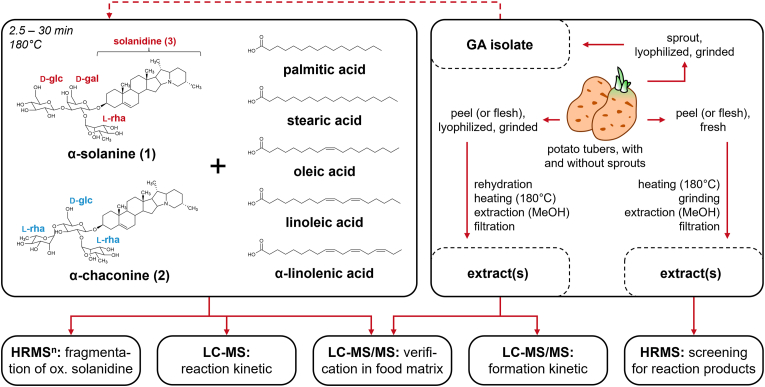
Overview of the experiments to investigate the reactivity of GA with fatty acids and validate their occurrence in a heated potato matrix. For model experiments, α-solanine and α-chaconine were heated with selected individual fatty acids (c.f. sections 3.2, 3.3 and 3.4). For some model experiments, GA were extracted and isolated from potato sprouts. For HRMS experiments (c.f. section 3.1), fresh potato peel was heated and used for a screening, while for LC-MS/MS experiments (c.f. section 3.5), freeze-dried potato peel powder was rehydrated with water and heated to verify reaction products in a potato matrix.

Glycoalkaloids (GA) are compounds mainly formed in the tissues of plants in the *Solanaceae* family such as potatoes (*Solanum tuberosum*), tomatoes (*S. lycopersicum)*, or eggplants (*S. melongena*) (Ammar et al., 2025; Martínez-García et al., 2026; Friedman, 2006, 2015; Zhao et al., 2021). The most prominent representatives of GA in potatoes are α-solanine (**structure 1**, Fig. 1) and α-chaconine (**2**, Fig. 1). These are typically concentrated in the skin and the layer beneath, with lower levels present in the flesh. During sprouting, GA synthesis is enhanced in the tubers, particularly in the developing sprouts. However, potatoes’ GA are quite well-known and characterized with regard to their potential toxicological effects, which can become significant when green, sprouting potatoes or large amounts of potato peel are consumed (Friedman, 2006; Milner et al., 2011;European Food Safety Authority, 2020).

α-Solanine and α-chaconine are composed of the same lipophilic aglycone, solanidine (**3**, Fig. 1), with different trisaccharides. α-Solatriose is composed of l-rhamnose, d-glucose, and d-galactose. In contrast, α-chacotriose is composed of two l-rhamnose units and d-glucose (Friedman, 2006; Kuhn et al., 1955a, 1955b; Zhao et al., 2021). As these chemical structures contain a polar and a non-polar moiety, amphiphilicity is mainly responsible for their bioactivity in the different organisms (Milner et al., 2011). The related compounds containing smaller saccharides are referred to as β_1_- and β_2_-GA, when one sugar moiety is hydrolyzed, and as γ-GA, when both outer sugar moieties are hydrolyzed, typically under acidic conditions (Friedman et al., 1993). In addition to hydrolytic cleavage resulting from processing, these compounds also can be found in small concentrations as natural intermediates of the biosynthesis (Baur et al., 2021; Cárdenas et al., 2015). Some studies have already shown that the hydrolysis products seem to have a lower bioactivity than the α-GA (Milner et al., 2011; Rayburn et al., 1994).

While it is well known that the GA content of food can be lowered by peeling (mechanical removal) and boiling (leaching into the water) the tubers (Mäder et al., 2009a, 2009b), an appropriate choice of potato cultivar and proper storage can also keep the GA content low (Petersson et al., 2013). However, less is known about GA's chemical degradation during further food processing procedures, especially at higher temperatures like frying of hash browns or deep-frying of French fries or potato crisps. Especially in the latter processes, temperatures of about 170 to 180 °C are realized and slices are usually deep-fried for 3 to 5 min (Arslan et al., 2018). The amount of scientific data on the decrease of α-GA during deep-frying is sparse. A decrease resulting from frying processes varies from almost 0% to more than 80% in the fat-free dry matter as reported in the literature (European Food Safety Authority et al., 2020; Martínez-García et al., 2025; Raters et al., 2024; Rytel, 2012; Rytel et al., 2018; Tajner-Czopek et al., 2014). These scarce but controversial reports led to a debate about GA stability during heating. However, the fate of degraded GA during potato processing has not yet been comprehensively resolved. To the best of our knowledge, no studies have reported specific chemical conversion pathways leading to products formed during the processing of potatoes into major food products, including crisps or French fries. Beyond the assumption that thermally-induced hydrolysis may occur, no additional reaction products, such as fragments or adducts, have been reported to date. This is consistent with the risk assessment of the EFSA, which stated that “no information has been found about the chemical nature of the GA degradation products” (European Food Safety Authority et al., 2020).

Generally, various endogenous compounds of the tuber could theoretically react with GA at elevated temperatures, providing a variety of options. A plausible source of reaction partners for GA may be lipids, as GA are considered to be present in the cell membranes. Due to their amphiphilic character and complex formation with sterols (Keukens et al., 1995, 1996; Roddick and Rijnenberg, 1986), it is considered possible that GA interact with the (phospho)lipids of the cell membrane. This mechanism – the incorporation of GA into the cell membrane – is incidentally the reason for its toxicity in mammals. However, the total lipid content of potato tubers is typically low, ranging from 0.1 to 0.2% of fresh weight and only rarely reaching up to 0.5%, with lipids predominantly localized in the peel and vascular tissues (Ramadan and Oraby, 2016). The concentration of free fatty acids in fresh tubers is correspondingly low and is generally reported in the range of a few micrograms per gram of fresh matter, approximately 2 to 4 μg per g fresh weight, representing only a minor fraction of total fatty acids, typically about 1% or less (Spychalla and Desborough, 1990). Lilja and Lingert (1989), however, showed that free fatty acid levels may increase substantially during processing, reaching up to approximately 25 μg per g fresh weight as a result of tissue disruption and lipase activity.

Based on this assumption, the central hypothesis of the present study was that GA are prone to heat-induced conversion, in general. Furthermore, it is hypothesized that they can react with endogenous compounds of the potato matrix – particularly amphiphilic compounds such as phospholipids or fatty acids. When frying or deep-frying, the frying oil is another apparent source of lipids that might be reaction partners for GA. Therefore, it is also hypothesized that frying oil influences the formation of lipid reaction products. Consequently, the study aimed at characterizing the stability of α-GA and their reactivity in heat-induced reactions as well as the formation of reaction products in a stripped model approach with free fatty acids, including palmitic, stearic, oleic, linoleic, and α-linolenic acid. Subsequently, the prevalence of reaction products is being investigated in heated potato matrices. Given that a higher amount of GA and lipids are present in the skin and the layer beneath in comparison to the flesh of the tubers, it was expected that a higher quantity of GA-derived reaction products could be found in potato peels and products with residual peel. To test all these hypotheses, heated potato peels are screened for reaction products by high resolution mass spectrometry. After characterizing potential reaction products in model experiments, potato samples are subsequently analyzed for the presence of reaction products that are identified in the model experiments, using targeted LC-MS/MS methods (Fig. 1). To evaluate the role of frying oil, subsequent experiments using potato matrices heated with sunflower oil were conducted, too.

In the present study, the primary aim was the identification of novel products, while toxicological risk assessment of potential degradation or reaction products was not the focus. Only when the chemical structures are fully resolved, and potential compounds are available as isolated compounds, follow-up experiments, such as quantification and toxicological risk assessment can be conducted and subsequently a valid risk communication strategy can be developed.

## 2 Material and methods

In a first step, potato peels, which are rich in GA, were heated and screened for potential reaction products (c.f. section 3.1). Afterwards, model systems consisting of the pure α-GA α-solanine and α-chaconine with selected fatty acids were established and heated at 180 °C (Fig. 1). Subsequently, multi-stage mass spectrometry experiments were performed to get insights into the chemical structure of the reaction products (c.f. section 3.2). Additionally, the formation of the reaction products was characterized using LC-MS (c.f. sections 3.3 and 3.4) and the presence of these reaction products has been verified in different potato matrices via LC-MS/MS in multiple reaction monitoring (MRM) mode (c.f. section 3.5).

### 2.1 Chemicals

α-Solanine and α-chaconine were purchased from PhytoLab GmbH & Co. KG (Vestenbergsgreuth, Germany). Acetonitrile (hypergrade) and formic acid (LC-MS grade) were purchased from Merck KGaA (Darmstadt, Germany). Methanol (HPLC grade), acetonitrile (HPLC grade), dichloromethane and chloroform were purchased from VWR International GmbH (Darmstadt, Germany). Palmitic acid, stearic acid, oleic acid, linoleic acid, and α-linolenic acid were purchased from TCI Deutschland GmbH (Eschborn, Germany). Phosphoric acid (85%) was purchased from Bernd Kraft GmbH (Duisburg, Germany).

### 2.2 Samples

For GA extraction, potatoes (*Solanum tuberosum*, cultivar (cv.) ‘Linda’) were purchased from a local supermarket and stored for some days at room temperature in daylight until sprouting started. Only the GA-rich sprouts were used for extraction. At that stage, selection of cultivar was only by availability in the supermarket. The potatoes (cv. ‘Agria’) for the further potato matrix experiments were purchased from local farmers shop near Berlin, Germany, shortly after harvesting. They were stored in a cool, dark place for a few days before being used in the lab. The tubers were neither greened nor sprouted. Cv. ‘Agria’ was chosen, because it is widely used for producing French fries – a process involving high temperatures. This cultivar is characterized by a low sugar content (Abbasi et al., 2011) and average amounts of lipids (0.22% of fresh matter, Abbasi et al., 2011), as well as GA (39 – 110 mg/kg fresh matter, Abbasi et al., 2011; Kasnak and Artık, 2018; Najm et al., 2012; Urban et al., 2018).

### 2.3 Isolation of a glycoalkaloid standard

The extraction and isolation of GA was performed according to Kozukue et al. (1999) with slight modifications. An additional purification step was added, and higher quantities of samples and solvents were used. Briefly, 12 g of lyophilized, green sprouts of approximately 1 cm length were ground (IKA Multidrive C S000, IKA-Werke GmbH & CO. KG, Staufen, Germany) and extracted three times in a mixture of 150 mL chloroform/methanol (2:1, v/v) at room temperature for 30 min with ultrasonic treatment. The organic extracts were combined, filtered (Büchner funnel; grade MN 615, 110 mm, Machery-Nagel GmbH & Co. KG, Düren, Germany), and the solvent was removed by rotary evaporation (IKA RV10 auto, IKA HB digital, IKA Vacstar digital, IKA-Werke GmbH & CO. KG, Staufen, Germany). The residue was taken up in 100 mL hydrochloric acid (0.2 M) using ultrasonic treatment at room temperature for 30 min (Elma Transsonic 460 H, Elma Schmidbauer GmbH, Singen, Germany) and filtered. The corresponding solution was alkalized with 30 mL ammonia solution (25%), incubated for 60 min at 60 °C, and then stored at −30 °C overnight. The precipitate was separated by centrifugation (2700 × g, Eppendorf 5804, Eppendorf SE, Hamburg, Germany) and washed three times with 10 mL ammonia solution (5%) in a Büchner funnel. The washed precipitate was dried completely by (rotary) evaporation. Green impurities were rinsed with small volumes of dichloromethane, while most of the GA did not dissolve. The white residue contained 96 ± 4% α-solanine and α-chaconine in a ratio of 38:62±3 (Fig. S6), which was determined using HPLC-UV (c.f. section 2.8) in comparison to commercially available reference compounds. In the extract, also β-GA, γ-GA, and the aglycone solanidine could be detected.

### 2.4 Experiments with potato matrix

For the screening of potential reaction products by HRMS (c.f. section 3.1), fresh potatoes (cv. ‘Agria’, c.f. section 2.2) were cleaned with water and peeled manually. The peels were heated in an oven (Heraeus UT 6, Heraeus Holding GmbH, Hanau, Germany) at 180 °C for 5 min, homogenized (IKA Multidrive C S000, IKA-Werke GmbH & CO. KG, Staufen, Germany), and 50 mg were extracted with 1 mL of methanol using ultrasonic treatment for 60 min. The resulting extract was filtered through a syringe filter (PTFE, 0.45 μm, 13 mm, VWR international LLC, Radnor, PA, USA).

For the verification of the compounds by LC-MS/MS (c.f. sections 3.3 and 3.4), fresh potatoes (cv. ‘Agria’, c.f. section 2.2) were cleaned with water and peeled manually. The peels and the flesh were frozen at −30 °C. Subsequently, the samples were lyophilized (Christ LOC-1M, Alpha 2–4 unity, Martin Christ Gefriertrocknungsanlagen GmbH, Osterode, Germany) and ground. 50 mg of the sample were transferred into an unsealed glass vial and 100 μL of water were added for rehydration. After an incubation for 1 h at room temperature, vials were heated for 2.5, 5, 10, 15, 20, 30, 45, 60, and 120 min in the oven at 180 °C. The dry residues were cooled down to room temperature and subsequently extracted with 1 mL of methanol using ultrasonic treatment at room temperature for 60 min. Prior to the LC-MS/MS measurements, samples were filtered using a syringe filter. The process of freeze-drying the samples and then reconstituting the resulting powder with water was used to prepare uniform samples in order to demonstrate the formation of reaction products at different heating times in triplicate.

For simulating the influence of a frying oil (Fig. S4 and Fig. S5), 50 mg of lyophilized ground potato peel were transferred into a glass vial and 100 μL of water were added for rehydration. After an incubation for 1 h at room temperature, 25 μL of sunflower oil was added and the glass vial was heated for 30 min at 180 °C and extracted and measured as described before.

### 2.5 Preparation of model experiments

For the model systems, described in section 3.2, either 25 μg of the α-solanine standard (28.8 nmol) or 25 μg of the α-chaconine standard (29.4 nmol) were dissolved in 50 μL methanol and mixed with 85 μg stearic acid (300 nmol in 50 μL *tert*-butyl methyl ether). The reaction mixtures were heated in an unsealed glass vial for 5 min at 180 °C. After heating, the samples were cooled down in a metal rack at room temperature and subsequently extracted with 1 mL of methanol with ultrasonic treatment for 30 min.

For the model systems in sections 3.3, 3.4 and 3.5, 50 μg of the isolated GA mixture were dissolved in 50 μL methanol (22.3 nmol α-solanine, 33.5 nmol α-chaconine, and approximately 5 nmol other GA, such as β-GA, γ-GA and solanidine). GA were either mixed with 50 μL (600 nmol in *tert*-butyl methyl ether) of one fatty acid, palmitic acid (153.8 μg), stearic acid (170.7 μg), oleic acid (169.5 μg), linoleic acid (168.3 μg), and α-linolenic acid (167.1 μg), or 50 μL (200 nmol) tristearate (178.3 μg). The reaction mixtures were heated in an unsealed glass vial for 2.5, 5, 7.5, 10, 15, 20, and 30 min at 180 °C. For individual treatment of GA without lipids, 50 μL of pure *tert*-butyl methyl ether was used with 50 μg of the GA mixture. After heating, the samples were cooled down in a metal rack at room temperature and subsequently extracted with 1 mL of methanol with ultrasonic treatment for 30 min. Obviously, the molar ratios of GA and fatty acids do not represent the original ratios in the potato. Instead, these molar ratios were chosen to specifically generate reaction products in the model system. In fact, free fatty acids are present in potatoes only in significantly smaller amounts (2 to 4 μg per g fresh weight; Lilja and Lingert, 1989; Spychalla and Desborough, 1990).

### 2.6 High-resolution mass spectroscopy (HRMS) for the characterization of novel compounds

HRMS analyses were performed in positive ion mode with a Thermo Fisher Scientific LTQ Orbitrap XL™ ion trap mass spectrometer equipped with an Ion Max ESI Source (Thermo Fisher Scientific Inc., Waltham, MA, USA). Samples were dissolved in methanol. The diluted reaction mixtures were infused using a syringe pump at a flow rate of 15 μL/min. Reserpine (0.05 mg/mL) was used as standard for mass calibration. For multi-stage fragmentation (MS^n^), the collision-induced dissociation energy (CID) was 40 for fragmentation of oxidized and non-oxidized solanidine (Fig. 3A and B) and 55 for the chaconine stearic acid ester and oxidized α-solanine (Table S1A and S1B). For the interpretation of the mass spectra, the software Freestyle 1.8 SP2 QF1 (Thermo Fisher Scientific Inc., Waltham, MA, USA) was used.

### 2.7 Hydrophilic interaction liquid chromatography coupled to tandem mass spectrometry (HILIC-MS/MS-ESI(+))

The analysis of the model systems as well as the models with food-like matrix were performed by liquid chromatography coupled with mass spectrometry on an Agilent Infinity 1260 High Performance Liquid Chromatography (HPLC) system equipped with an Agilent G6470A Triple Quad mass spectrometer detector coupled to an electrospray ionization source (ESI) in positive mode (Agilent Technologies Inc., Santa Clara, CA, USA).

Separation was performed on a XBridge™ BEH Amide Column (3.5 μm, 130 Å, 250 × 4.6 mm, Waters Corp., Milford, MA, USA) and the following settings were used: column temperature, 30 °C; flow rate, 0.8 mL/min; eluent A, 0.1% formic acid in water (v/v); eluent B, acetonitrile; 0 min, 10% A; 2 min, 10% A; 12 min, 50% A; 18 min, 50% A; 20 min, 10% A, 5 min post run; injection volume, 2 μL; desolvation gas, nitrogen; desolvation gas temperature, 200 °C; gas flow rate, 11 L/min; nebulizer pressure, 35 psi; sheath gas temperature, 275 °C; sheath gas flow, 11 L/min. Detection was achieved by multiple reaction monitoring (MRM) as well as selected ion monitoring (SIM) with *m/z* of the proton adduct [M+H]^+^ of each analyte (ion mass in SIM mode and precursor mass for MRM mode). For MRM mode, *m/z* 98.1 was chosen as product ion for all non-oxidized analytes and *m/z* 120.1 for all oxidized analytes. The ions with *m/z* 98.1 and 120.1 were selected because they were the fragment ions with the highest intensity. Additionally, they were formed only in native or oxidized GA, respectively. They can therefore be used to differentiate native and oxidized GA. The fragmentor voltage was 150 V and the CID was individually chosen between 50 and 170 V. Additional information about the SIM and the MRM method can be found in Table S2 in the Supplementary Data.

### 2.8 HPLC-UV of α-glycoalkaloids

Analysis of α-solanine and α-chaconine by HPLC-UV was performed according to Distl and Wink (2009) with slight modifications of the eluent gradient. The following HPLC setup was used (Agilent Technologies Deutschland GmbH, Waldbronn, Germany): pump, G1312A; degasser, G1322A; autosampler, G1313A; column oven, G1316A; detector G1313A; column, Nucleosil™ 120-5 C18 EC 5 μm 250 × 4 mm (MACHEREY-NAGEL GmbH & Co. KG, Düren, Germany); software, MassHunter Workstation 10.1, MassHunter Qualitative Analysis Navigator 8.00, MassHunter Quantitative Analysis 8.0. The following settings were used: column temperature, 35 °C; flow rate, 0.75 mL/min; eluent A, 0.0425% phosphoric acid in water (v/v); eluent B, acetonitrile; eluent gradient, 0 min, 70% A; 1 min, 70% A; 25 min, 64% A; 29 min, 20% A; 34 min, 20% A; 38 min, 70% A; 43 min, 70% A, post time, 2 min; wavelength for quantitation, 202 nm. For this method, a LOD of 1 μg/mL and a LOQ of 5 μg/mL were determined.

### 2.9 Statistical analysis

**Fig. 2:**
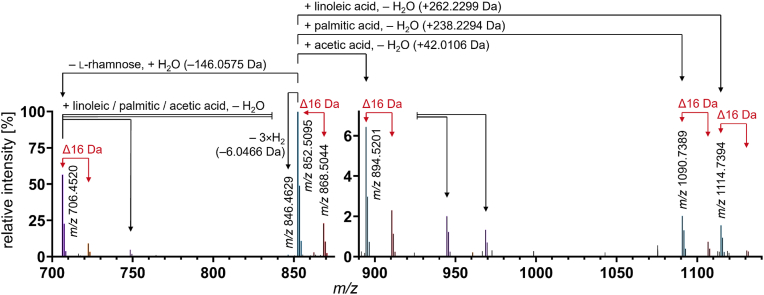
High-resolution mass spectrum of potato peels after heating for 5 min at 180 °C. The recurring mass difference of 15.9949 Da is marked with “Δ16 Da” and corresponds to the difference of one oxygen atom. The proposed reactions of α-chaconine (*m/z* 852.5095) are shown by black arrows: hydrolysis of rhamnose (−146.0575 Da, *m/z* 706.4520, same *m/z* as β_1_-solanine), oxidation (−6.0466 Da, *m/z* 846.4629), or esterification with either acetic acid (+42.0106 Da, *m/z* 894.5201), palmitic acid (+238.2294 Da, *m/z* 1090.7389), or linoleic acid (+262.2299 Da, *m/z* 1114.7394).

The semiquantitative data in Fig. 2, Fig. 5, Fig. 8, and Fig. S3 were prepared and analyzed in triplicate. All results are shown as means ± standard deviation. Significant differences (p < 0.05) were analyzed by one-way analysis of variance (ANOVA) and TUKEY's test using the Prism 8.0.2 software (GraphPad Software Inc., San Diego, CA, USA).

## 3 Results and discussion

### 3.1 HRMS characterization of reaction products in potato peels

For characterizing the potential conversion of GA during processing of potato crisps and French fries, direct-infusion HRMS was employed to monitor the formation of smaller GA β- and γ-derivatives and reaction products. Fig. 2 shows a mass spectrum of the methanolic extract of potato peels simply heated without addition of an external (frying) oil. As potato peels generally have a higher GA content than potato flesh, it was assumed that more reaction products are present after a heat treatment. Using electron-spray ionization in positive ion mode, the most abundant signals were assigned to molecules containing one nitrogen atom based on their exact mass. The majority of these were protonated GA and GA reaction products.

**Fig. 3:**
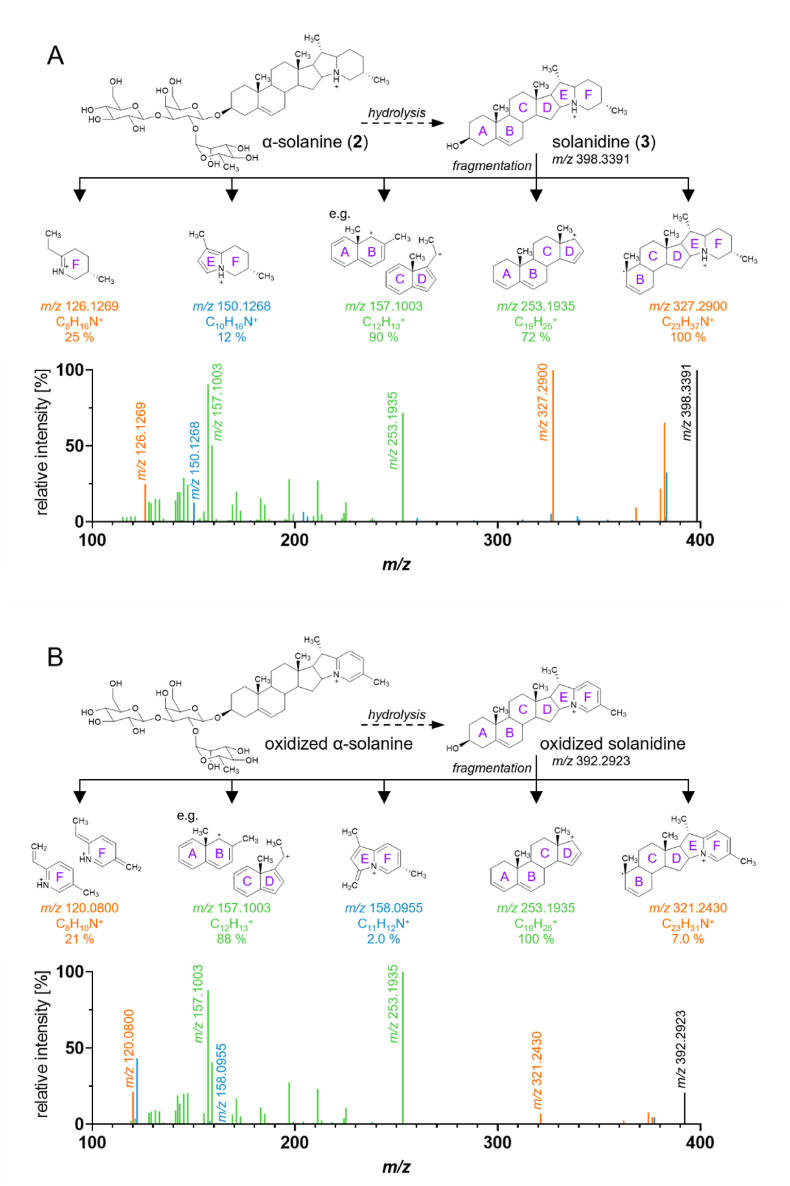
Multi-stage high-resolution mass spectra of **A)** solanidine (*m/z* 398.3391) and **B)** oxidized solanidine (*m/z* 392.2923, tentative structure suggestion) in a reaction mixture of α-solanine and stearic acid after 20 min of heating at 180 °C. Full fragment list in Table S3. Group I: green; group II: orange; group III: blue.

The most intense signal was assigned to protonated α-chaconine ([M+H]^+^ *m/z* 852.5095, defined as 100%). α-Solanine tends to form single ([M+H]^+^, *m/z* 868.5044) and double charged ions ([M + H + Na]^2+^, *m/z* 445.7468; Fig. S1) with a lower signal of *m/z* 868.5044.

The mass difference of 15.9949 Da (“Δ16 Da”, Fig. 2) between α-solanine and α-chaconine is a helpful marker for differentiation. This value is based on one oxygen atom and is a result of the glycosidic rest of α-chaconine containing two 6-deoxy sugar moieties (**2,** Fig. 1), while α-solanine only carries one (**1,** Fig. 1). This difference was also found between signals of the well-known hydrolysis products *m/z* 706.4520 (for β_1_-chaconine, β_2_-chaconine, and β_1_-solanine) and *m/z* 722.4469 (for β_2_-solanine).

In the range of *m/z* 890 to 1150 (right diagram, Fig. 2), signals of unknown composition with an intensity below 7% were detected, that also differed in their *m/z* by one oxygen atom (*m/z* 894.5201 and 910.5150, 1090.7389 and 1106.7342 as well as 1114.7394 and 1130.7344). Based on their exact mass, the difference between the α-GA and these new compounds were tentatively assigned to the chemical formulae C_2_H_2_O (Δ*m/z* 42.0106), C_16_H_30_O (Δ*m/z* 238.2296), and C_18_H_30_O (Δ*m/z* 262.2299), which are most likely condensation products with organic acids. Due to the exact masses and their prevalence in potato lipids (Galliard, 1973), palmitic acid (15-20% of total fatty acids) and linoleic acid (50-61%) were suspected to be main conjugates. Similarly, the signals *m/z* 894.5201 and 910.5150 were assigned to esters of α-GA with acetic acid. Analogous reaction products were detected for β_1/2_-chaconine and β_1_-solanine (*m/z* 706.4520 → 748.4625, 944.6814, and 968.6813), β_2_-solanine (*m/z* 722.4469 → 764.4573, 960.6768, and 984.6750), and γ-GA (*m/z* 560.3941 → 602.4048, 798.6235, and 822.6232). The subsequent verification of these reaction products was provided by follow-up model experiments and LC-MS/MS analysis as described in sections3.3, 3.4 and 3.5.

In addition to the aforementioned ester formation, further derivatives of the native GA were detected. Some of these were hypothesized to be triple oxidized α-chaconine and oxidized α-solanine, because of the signals at *m/z* 846.4629 and 862.4576, which differ by 6.0466 Da and 6.0468 Da from α-chaconine and α-solanine, respectively.

### 3.2 Structural elucidation of oxidized solanidine by HRMS^n^

To gain further insight into the chemical structure of the esterified and oxidized GA, the sample extract obtained by heating α-solanine and α-chaconine with stearic acid for 5 min at 180 °C, respectively, was analyzed using multi-stage high-resolution mass spectrometry (HRMS^n^). The fragmentation patterns of the α-chaconine stearic acid ester and oxidized α-solanine are given in Table S1A and S1B, respectively. During the fragmentation of the α-chaconine stearic acid fatty acid ester, the ester could only be detected while sugars remained attached (e.g., the β-chaconine stearic acid ester, *m/z* 972.7145), indicating that the esterification occurs at the sugar moiety. During the fragmentation of the oxidized α-solanine, the oxidized structures remained on the fragment ions despite sugar hydrolysis. Oxidized β-solanines (*m/z* 716.4006 and *m/z* 700.4006), γ-solanines (*m/z* 554.3476) and solanidine (*m/z* 392.2949) were detected as fragments, indicating that the oxidation occurs on the steroidal alkaloid moiety.

To characterize the structure of the oxidized steroidal moiety, *m/z* 398.3391 (solanidine, Fig. 3A) and *m/z* 392.2923 (oxidized solanidine, Fig. 3B) were fragmented further. In theory, this fragmentation can be studied either through tandem experiments involving the fragmentation of, for example, native (m/z 868.5044 → 398.3391 →) and oxidized (m/z 862.4585 → 392.2923 →) α-solanine, or directly from native (*m/z* 398.3391 →) and oxidized (*m/z* 392.2923 →) solanidine, which is formed by hydrolysis in the model described above. As the intensities of the solanidine derivatives formed by hydrolysis are higher and the exact mass can be determined with a higher degree of certainty, these were selected for interpretation. For interpretation of the fragmentation pattern, the ions in Fig. 3 were sorted into three groups (full fragment list in Table S3): I) ions that do not contain nitrogen and are present in the spectra of the oxidized and the non-oxidized aglycone (Fig. 3A and B, green); II) pairs of ions that are formed only from the oxidized (Fig. 3B) or non-oxidized solanidine (Fig. 3A), respectively, and which also differ by 6 Da, all of them containing one nitrogen atom (orange); III) ions that do neither belong to group I nor II and are mostly formed during the fragmentation of either the oxidized or the non-oxidized solanidine (Fig. 3A and B, blue).

The ions at *m/z* 157.1003 and 253.1935 were present in both fragment ion spectra (Fig. 3A and B) with a high abundancy, do not contain nitrogen, and therefore belong to group I. Their composition was assigned to C_12_H_15_^+^ and C_19_H_25_^+^, respectively. Besides these two fragment ions, there were further fragment ions that solely consist of hydrocarbons and therefore also belong to group I (Table S3). Most of these hydrocarbon fragments can be found in both spectra in similar intensities. This gives rise to the assumption that the oxidation is not located in the rings A, B, C, and D, even though there is already an unsaturated C-C-bond.

The ions at *m/z* 321.2430 and 327.2900 as well as *m/z* 120.0800 and 126.1269 are pairs of ions that are formed only from the oxidized (Fig. 3B) or non-oxidized solanidine (Fig. 3A), showing a mass difference of 6 Da. Given that they are nitrogen-containing fragments, they can be assigned to group II. It was assumed that this mass difference results from the oxidation. As these four fragment ions all contain nitrogen, oxidation seems to take place in the vicinity of the hetero atom – either in the E or the F ring.

For the ions at *m/z* 321.2430 and 327.2900, a fragmentation of the A ring was proposed for both structures, leaving behind an unpaired electron that is stabilized by the unsaturated C-C-bond at the B-ring. The ion of *m/z* 126.1269 was already described by Cahill et al. (2010) during fragmentation of non-oxidized solanidine, consisting of the F ring. Analogously, a fragment with *m/z* 120.0800 was formed during fragmentation of the oxidized solanidine, whose *m/z* is 6 Da smaller. Based on the structural proposal for Cahill's fragment, a pyridine-like structure is proposed for this fragment, but with an oxidized F ring.

When considering the position of the double bonds, also energetic properties of the oxidized solanidine must be taken into account, as a more energetically stable product would be preferred. Due to the cyclic structure, the formation of an aromatic compound, whose π-electron valence must comply with the Hückel's rule, is strongly expected. The three double bonds can be arranged in the six-membered F ring (piperidine derivative), resulting in the formation of a pyridinium derivative in accordance with the Hückel's rule. In contrast, in the five-membered E ring (pyrrolidine derivative), only two π-bonds are required to form an aromatic ring (pyrrole derivative). The third double bond/oxidation would have to occur at another point in the molecule, independent of the aromatic ring. This would mean that there would have to be double oxidized derivatives. As there is no evidence of a doubly oxidized solanidine, it is tentatively proposed that the triple oxidized solanidine has an aromatic F-ring.

This suggestion was supported by the formation of *m/z* 158.0955 of the oxidized solanidine and *m/z* 150.1268 of the non-oxidized solanidine. Both fragment ions contain the E− and the F-ring, resulting from a fragmentation of the D-ring. For *m/z* 150.1268, a pyrrole structure of the E ring is proposed (Fig. 3A). In contrast, the formation of a fragment with a similar pyrrole structure resulting from the oxidized solanidine seemed to be thwarted by the hypothetical oxidation of the F-ring. Instead, the formation of the fragment ion at *m/z* 158.0955 is favored with the molecular structure proposed in Fig. 3B. Beyond, the fragment ion at *m/z* 98.1 is strongly favored during the fragmentation of non-oxidized solanidine. To prevent discrimination of other fragments, the range in these HRMS^n^ experiments was limited to *m/z* 100 to *m/z* 392 and 398, respectively. The fragment of *m/z* 98.1 could not be found in the fragmentation of oxidized solanidine (not shown), which supported the assumption of an oxidized F-ring. However, in the LC-MS/MS analysis, the product ion at m/z 98.1 was selected for all non-oxidized GA, while the product ion at m/z 120.1 was selected for oxidized GA.

Due to the complex mixture of reaction products, subsequent isolation is a difficult task. Thus, it cannot be within the scope of the present study yet to isolate the mentioned reaction products and to unambiguously elucidate their chemical structure. Instead, the chemical structures have been tentatively identified and further studies should focus on providing the necessary additional evidence by nuclear magnetic resonance (NMR) spectroscopy.

### 3.3 Chromatographic separation and mass spectrometric detection of α-GA and their reaction products in model systems

**Fig. 4:**
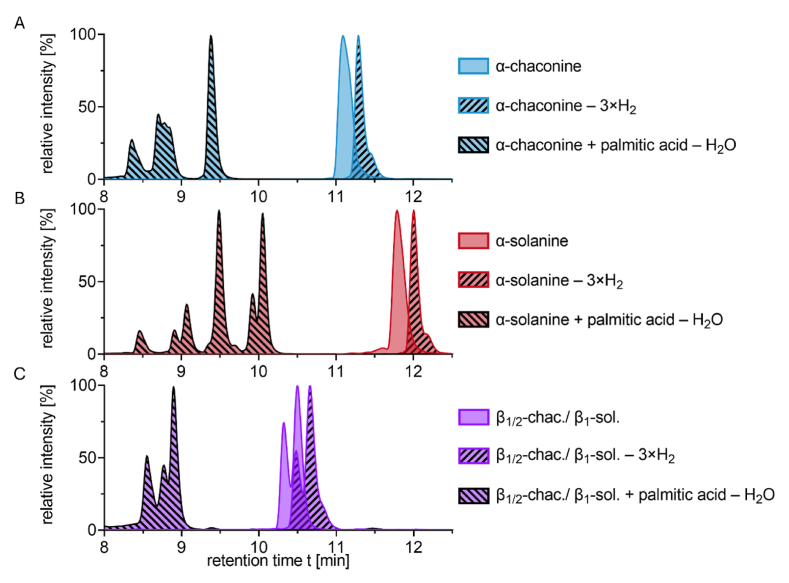
Chromatograms of reaction products of α-solanine and α-chaconine with palmitic acid, heated for 5 min at 180 °C. **A)** α-chaconine (*m/z* 852.5 → 98.1), triple oxidized α-chaconine (*m/z* 846.5 → 120.1), and palmitic acid α-chaconine ester (*m/z* 1090.7 → 98.1); **B)** α-solanine (*m/z* 868.5 → 98.1), triple oxidized α-solanine (*m/z* 862.5 → 120.1), and palmitic acid α-solanine ester (*m/z* 1106.7 → 98.1), **C)** β_1_-chaconine, β_2_-chaconine and β_1_-solanine (*m/z* 706.5 → 98.1), triple oxidized β_1_-chaconine, β_2_-chaconine and β_1_-solanine (*m/z* 700.5 → 120.1), and palmitic acid esters of β_1_-chaconine, β_2_-chaconine and β_1_-solanine (*m/z* 944.7 → 98.1).

For a deeper characterization of the novel compounds, model experiments were conducted by heating α-solanine and α-chaconine with the ten times molar ratio of the fatty acids of interest. In Fig. 4 chromatograms of selected reaction products of the model system with palmitic acid after 5 min heating are shown.

In the model systems, all hydrolysis products (e.g., β_1/2_-chaconine, γ-chaconine, and solanidine) were detected. In addition, the proposed esters (e.g., α-chaconine + palmitic acid – H_2_O) and oxidation products of all hydrolysis products (e.g., α-chaconine – 3×H_2_) were found as well (c.f. section 3.4). Analogous compounds were found for α-solanine (e.g., α-solanine + palmitic acid – H_2_O) (Fig. 4).

The retention time as well as the number of peaks provide important information about the structural properties of the compounds. Given that a HILIC column was used for the chromatographic separation of the analytes, polar α-GA eluted later than the less polar β-GA or the GA fatty acid esters. The higher retention time of the oxidized GA in comparison to the native GA provides two conclusions (Fig. 4A). First, it can be excluded that the oxidized GA are artifacts only formed during ESI, because otherwise the retention time would have been the same. Instead, they must be formed as a result of the heat treatment. Second, due to the higher retention time, the oxidized GA are slightly more polar than the native GA.

Regarding the esters of α-GA and palmitic acid, multiple peaks were detected. Considering that α-solanine has nine hydroxyl groups, up to nine potential constitutional isomers might be formed. In fact, the chromatogram showed at least six separate peaks (Fig. 4B). Similarly, the chromatogram of the palmitic acid α-chaconine esters showed five peaks (Fig. 4A). This difference in the number of hydroxyl groups and the number of peaks could be explained by the formation of isomers with a similar steric arrangement, such as the esters of α-solanine via the equatorial hydroxyl groups at C3 and C4 in l-rhamnose and C2, C3, and C4 in d-glucose with similar retention times each.

Two of the isomers with α-solanine were formed preferably in comparison to the other isomers, whereas only one isomer seems to be preferred the most with α-chaconine. This may be due to the primary hydroxyl group at C6 of d-glucosyl or d-galactosyl of which α-solanine has two and α-chaconine only one. For steric reasons, primary hydroxyl groups are better nucleophiles and therefore being more likely to form esters (Dimakos and Taylor, 2018). Horton and Lauterbach, 1969 incubated 1-methylglucose in equimolar ratio with acetic anhydride in pyridine at room temperature, the ratio of the acetylation at the four remaining hydroxyl groups was approximately 40% at C6, 20% at C4, 20% at C3, and 20% at C2. This underlines the results of the present study. Analogously to the α-GA, oxidized β-GA and at least three constitutional isomers of the β-GA palmitic acid esters were found, too (Fig. 4C).

### 3.4 Conversion of GA and formation of the reaction products in model systems

**Fig. 5:**
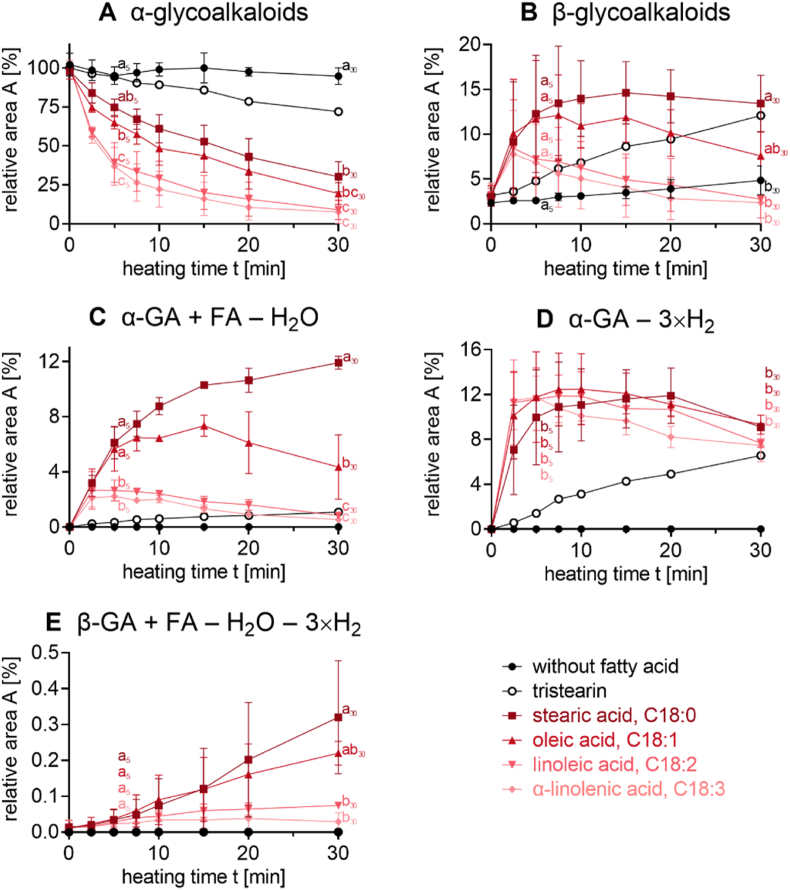
Conversion of α-GA and formation of reaction products with fatty acids (FA) stearic acid, oleic acid, linoleic acid, or α-linolenic acid, or individual treatment ('control', n = 3). **A)** α-GA (*m/z* 852.5 and 868.5), **B)** β-GA (*m/z* 706.5 and 722.4), **C)** ester of α-GA and corresponding fatty acids (*m/z* 1112.7 and 1128.7, *m/z* 1114.7 and 1130.7, *m/z* 1116.7 and 1132.7, *m/z* 1118.7 and 1134.7), **D)** triple oxidized α-GA (m/z 846.5 and 862.5), **E)** ester of triple oxidized β-GA and corresponding fatty acids (*m/z* 966.7 and 982.7, *m/z* 968.7 and 984.7, *m/z* 970.7 and 986.7, *m/z* 972.7 and 988.7). Statistical analyses were performed by one-way ANOVA and TUKEY's test (p < 0.05). Statistically equal values of the data points are designated by equal letters. Data points were grouped by heating time (comparison of the difference based on the fatty acid) and exemplarily given for 5 and 30 min. Complete statistical remarks can be found in Fig. S2 and the formation of further reaction products in Fig. S3.

To get insights into the formation mechanism of the novel reaction products, the degradation kinetics of the α-GA were determined in model systems with stearic acid (C18:0), oleic acid (C18:1), linoleic acid (C18:2), and α-linolenic acid (C18:3; Fig. 5) as well as stearin (glyceryl tristearate, Fig. S2 and Fig. S3), and individual heating without lipids. Additionally, the formation of the individual reaction products of GA was monitored (hydrolysis products, esters with fatty acids, oxidation products and 'mixed products', Fig. 5). In order to get a better overview, Fig. 5 andFig. 6 depict the sums of corresponding substances (e.g., α-chaconine and α-solanine as α-GA in Fig. 5A).

Individual treatment of pure α-GA ('control') did not result in a significant conversion of α-solanine and α-chaconine (Fig. 5A, Fig. S2 and Fig. S3A&B). Consequently, only a few hydrolysis products (Fig. 5B) and no other reaction products (Fig. 5C–E) were detected.

As fatty acids are mainly present as part of triacylglycerols in food systems, α-GA were initially incubated with stearin (Fig. S2, n = 1). In this reaction system, a moderate degradation of α-GA was observed, potentially due to the preceding hydrolysis of the glyceryl stearate ester and the subsequent reaction of the free fatty acid(s) with the GA.

Indeed, a higher conversion rate of α-GA was observed, when α-GA were incubated with fatty acids (Fig. 5A) and therefore used for the investigations. In these model systems, α-solanine and α-chaconine were converted with a similar reaction rate (Fig. S3A and B). However, the conversion rate was higher when polyunsaturated fatty acids were used instead of saturated fatty acids (Fig. 5A). E.g., after 30 min of heating, only 10% of the initial α-GA were detected after incubation with polyunsaturated fatty acids, whereas around 20% or 30% were still left in the binary systems of GA with oleic or stearic acid, respectively.

In the model systems with the free fatty acids, the hydrolysis products β-GA (sum of β_1_-solanine, β_2_-solanine, β_1_-chaconine, and β_2_-chaconine, Fig. 5B), γ-GA, and the aglycone solanidine (Fig. S3C and D) were present already at the beginning of the heating process. After 2.5 min, the respective concentrations of β-GA and solanidine increased at the same level in all model systems. However, in the systems with the polyunsaturated fatty acids, concentration of the hydrolysis products decreased again already after 5 min, whereas in the systems with saturated fatty acids, the concentration increased to an even higher level (Fig. 5B and Fig. S3D). The amount of γ-GA increased only in model systems with saturated and monounsaturated fatty acids but not with polyunsaturated fatty acids (Fig. S3C).

The formation of the individual α-GA fatty acid esters showed kinetics similar to those of the formation of β-GA (Fig. 5C). After 2.5 min, the concentration of each ester was nearly the same, while after 5 min, there was a further increase in the models with the saturated and the monounsaturated fatty acids and a decrease with polyunsaturated fatty acids. After 30 min of heating, there were more esters of α-GA and saturated and monounsaturated fatty acids than polyunsaturated fatty acids in the respective model system. In some cases, even diesters were identified (Fig. S3E). It could not be excluded that higher degrees of esterification occurred, too. However, concentrations are expected to be lower than the potential limit of detection. As mentioned in sections 3.1 and 3.3, esters of hydrolysis products can also be found. Similarly, the β-GA stearic acid ester is present in the largest quantity (Fig. S3I and J).

The formation of oxidized α-GA (Fig. 5D) was the fastest in the incubation mix with the polyunsaturated fatty acids. However, oxidation even took place in significant amounts when saturated fatty acids were applied. Similar to the esters and hydrolysis products (Fig. 5B and C), after 5 min there was a maximum of the concentration in the system with α-linolenic acid, whereupon the concentration decreased afterwards. However, incubations with different fatty acids did not differ significantly from each other. The oxidized form was found for all hydrolysis products, such as β-GA, γ-GA, and the aglycone (Fig. S3F–H). Similarly to the oxidized α-GA, concentrations of oxidized hydrolysis products were similar in model systems with the four individual fatty acids.

Reaction products that are oxidized and esterified at once were detected, too (Fig. 5E and Fig. S3K). In Fig. 5E, an exemplary reaction product, resulting from the three different reaction types is shown. A significant formation was only observed when saturated and monounsaturated fatty acids were used. However, the relative abundance of these analytes was low, presumably due to the multiple parallel reaction pathways and the large number of reaction products that are formed.

Fig. 5 displays relatively large error bars for several measurements, which may hinder the direct visual identification of significant differences. Data were obtained from triplicate experiments conducted on separate days. Within each replicate, samples with varying fatty acid systems and heating times were prepared simultaneously and subjected to identical analytical procedures. Despite strict standardization of heating and handling parameters, substantial variability remained. Nevertheless, all replicates and model systems showed consistent reaction profiles, with comparable qualitative outcomes, supporting the robustness of the qualitative trends. The variability was primarily associated with differences in maximum response intensity and apparent reaction rate. This behavior indicates that the reaction pathways involved are highly susceptible to subtle external influences, even under carefully controlled conditions.

In order to estimate the amount of reaction products formed, the analytes were measured in SIM mode. Assuming that all analytes are protonated at the steroidal nitrogen during electrospray ionization, a similar response for all analytes can be expected. Under this assumption, the integrated peak areas of different analytes can be compared. After 2.5 min of heating, the relative peak areas were 7–10% (Fig. 5B) for the β-GA, 2–4% (Fig. 5C) for the α-GA fatty acid esters, and 7–12% (Fig. 5D) for the oxidized α-GA. After prolonged heating, the total area of the reaction products in the model system containing stearic acid did not exceed 15%. Given the uncertainty regarding the exact response factors of the analytes in SIM mode, the amounts formed via the three pathways appear to be comparable. As the three pathways seem to proceed at similar rates, they likely contribute to a similar extent to the overall conversion of GA. From a mechanistic point of view, the three pathways occur independently of each other.

**Fig. 6:**
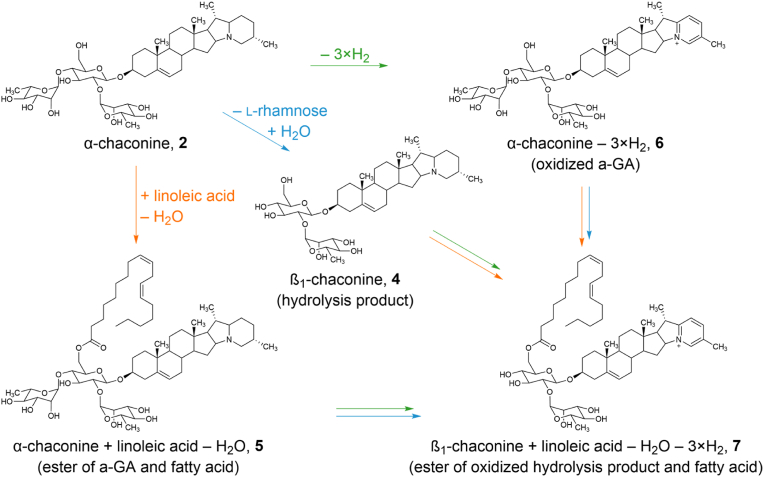
Chemical structures of α-chaconine (top left, **2**) and its three exemplary reaction products: a hydrolyzed product (β_1_-chaconine; center, **4**), an ester formed with linoleic acid (bottom left, **5**, tentative structural suggestion of the dominant constitutional isomer), the triple oxidized α-chaconine (top right, **6**, tentative structure suggestion), and in addition a proposed structure of the combination of the three reactions taking place, the triple oxidized β_1_-chaconine esterified with linoleic acid (bottom right, **7**, tentative structural suggestion of the dominant constitutional isomer).

**Fig. 7:**
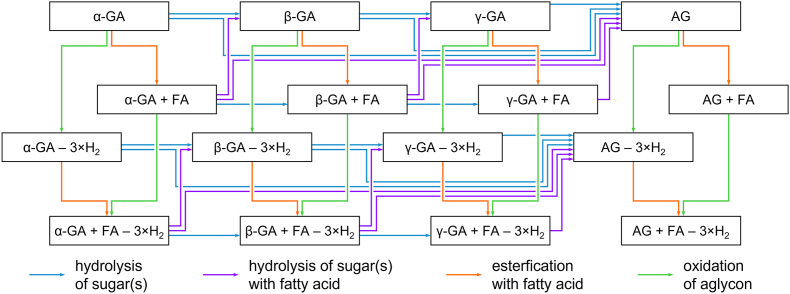
Scheme of the formation pathways of novel GA derivatives. The three main reactions are hydrolysis of a sugar (blue and purple), esterification with a fatty acid (FA) (orange), and oxidation of the aglycone (green). For clarity of presentation, diesters are not shown.

In Fig. 6, tentative structure suggestions of the before mentioned GA-derived reaction products, the hydrolysis product β_1_-chaconine (**4**), the dominant constitutional isomer of the ester of linoleic acid and α-chaconine (**5**), the oxidized α-chaconine (**6**), and a mixed compound, the dominant constitutional isomer of the ester of linoleic acid and oxidized β_1_-chaconine (**7**), resulting from a system containing α-chaconine (**2**) and linoleic acid are shown exemplarily. Furthermore, a multitude of other reaction products based on a combination of hydrolysis, oxidation, and esterification of GA was also identified in the model systems, given schematically in Fig. 7. It is apparent that different pathways yield the same products and can be encompassed consecutively or in parallel. Starting from α-GA, sugar hydrolysis can proceed in different steps, e.g., releasing single monosaccharides or the whole trisaccharide to form the β-GA or the aglycone, respectively (blue). The formation of γ-GA requires the formation of β_1_-or β_2_-GA first, as the two outer sugar moieties cannot be hydrolyzed in one step.

In theory, the fatty acid esters (“+ FA”) can be found for all GA as long as there are hydroxyl groups available – either from a sugar or the aglycone. Once formed, esters can also be hydrolyzed again, depending on individual stability and equilibrium of the whole set of reaction pathways (orange). As the esterification takes place at the glycosidic residues, the fatty acid esters can be co-eliminated by hydrolysis of the sugar (purple). Also, oxidation (“– 3×H_2_”) can occur at all GA, including solanidine, with and without fatty acid esters, as the oxidation takes place on a different part of the molecule in contrast to the esterification (c.f. section 3.2; green). While the esterification is a reversible process, the oxidation is assumed to be nearly irreversible due to the energetic stability. In addition, the oxidation can only occur once while the esterification can take place at multiple positions in the whole molecule.

As the three reactions described – hydrolysis, esterification, and oxidation – can appear in parallel, a large number of GA-derived reaction products is to be expected. Hence, each reaction product will only be formed in small quantities. As these reactions can occur simultaneously and seem to have a similar rate, the reaction pathway gets complex quite early and each fatty acid contributes to the complexity of this reaction through its own unique kinetics, even in simple reaction mixtures. In the case of the potato matrix, an even greater branching of paths is expected.

In general, initially induced conversion steps, such as the formation of α-GA fatty acid esters, appear to proceed at comparable rates for all fatty acids. This is consistent with the findings of Hartman (1966), who reported no significant differences in the formation rates of esters from glycerol and the four fatty acids used herein. It is assumed that the mechanism is based on a typical nucleophilic attack by the hydroxyl group of the sugar moiety on the electrophilic carboxyl group carbon of the fatty acid. However, differences become apparent in follow-up reactions, which proceed more rapidly in the presence of polyunsaturated fatty acids. As a result, intermediate reaction products tend to accumulate to a more pronounced extent in model systems containing saturated and monounsaturated fatty acids. Although detailed dynamics remain elusive, there could be, in theory, several factors contributing to the accelerated conversion of GA observed in systems containing polyunsaturated fatty acids. First, differences in acidity may play a role. Unsaturated fatty acids exhibit lower pKa values (stearic acid: 10.15; oleic acid: 9.85; linoleic acid: 9.24; α-linolenic acid: 8.28; Kanicky and Shah, 2002). This increased acidity could promote proton-catalyzed hydrolysis of GA. Second, radical-mediated processes are likely to be more pronounced in systems containing unsaturated fatty acids, because allylic hydrogen atoms in polyunsaturated fatty acids can be readily abstracted, particularly in the presence of oxygen, leading to the formation of lipid radicals, reactive oxygen species, and secondary radical intermediates (Choe and Min, 2006; Porter et al., 1995; Pratt et al., 2011; Tietel et al., 2023). These species may enhance the overall reactivity of the system.

Beyond lipid-related mechanisms, thermal treatment of GA can also lead to hydrolysis of the glycosidic moiety, releasing reducing sugars. These sugars may undergo further reactions, such as caramelization or maillard reaction (Hodge, 1953), resulting in the formation of reactive carbonyl compounds. Consequently, the model systems likely involve complex interactions between lipid-derived radicals, sugar degradation products, and oxygen in an acidic environment. Despite the plausibility of enhanced radical formation in systems containing polyunsaturated fatty acids, oxidized GA were detected in similar amounts in model systems with both saturated and polyunsaturated fatty acids. This indicates that lipid oxidation products are unlikely to be the primary drivers of GA oxidation. Instead, GA oxidation may proceed predominantly via intrinsic reaction pathways, or radical species may be consumed in competing side reactions, thereby limiting their net contribution to GA oxidation.

In summary, it can be expected that multiple, partially overlapping mechanisms contribute to GA degradation. While ester formation can be rationalized by typical acid-catalyzed reactions, the oxidation of GA likely involves a complex interplay of acid catalysis, and carbonyl-mediated transformations, possibly in combination with radical reactions. However, the faster decrease of α-GA in systems containing unsaturated fatty acids imply that additional reaction pathways, potentially involving reactive oxygen species, may be involved in GA degradation as well. At present, the available data do not allow a definitive mechanistic conclusion. Further investigations, for example using systems with isolated reaction intermediates, radical scavengers, glycoside-free educts or controlled oxygen availability and controlled pH levels, would provide valuable insight into the individual pathways and offer a more detailed understanding of the mechanisms involved in GA degradation.

### 3.5 Presence of the reaction products in food-like matrices

In the model systems, a wide variety of compounds were identified. To show their presence in real food-like matrices, heated potato peels were analyzed by HILIC-MS/MS in MRM mode with regard to precursor ions, fragment ions, and retention times of the compounds described before in the model systems. Therefore, a powder of freeze-dried potato peels was rehydrated and heated in an oven for up to 120 min at 180 °C.

**Fig. 8:**
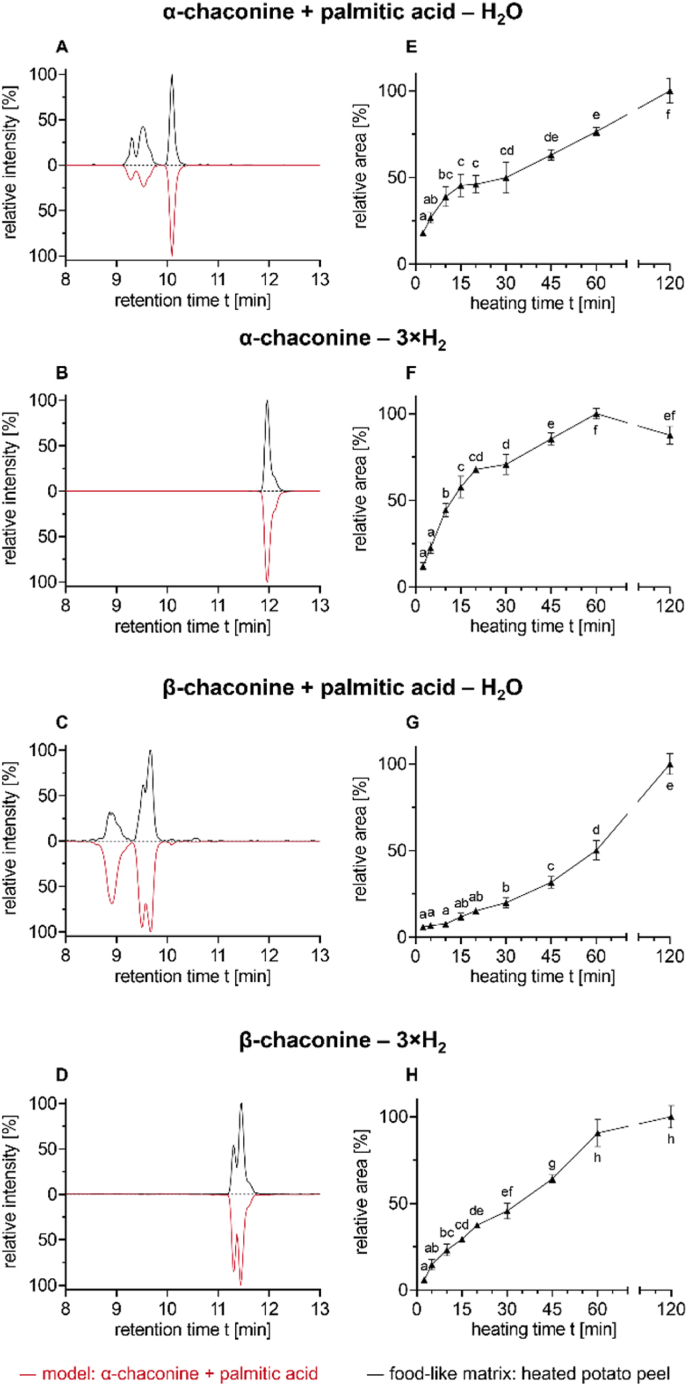
Formation of the reaction products in heated, rehydrated potato peel powder (black) compared to the model systems (red). **A-D)** Chromatograms of selected reaction products after 5 min of heating; **A)** palmitic acid α-chaconine ester (*m/z* 1090.7 → 98.1), **B)** triple oxidized α-chaconine (*m/z* 846.5 → 120.1), **C)** the esters of the β-chaconines and palmitic acid (*m/z* 944.7 → 98.1), and **D)** triple oxidized β-chaconine (*m/z* 700.5 → 120.1). **E-H)** Formation trend of the reaction products of Fig. 8A–D. Each diagram is individually normalized to 100%, based on the highest measured signal intensity. Statistical analyses were performed by one-way ANOVA and TUKEY's test (p < 0.05). Statistically equal values of the data points are designated by equal letters.

The presence of GA-derived reaction products in food-like matrices is displayed in Fig. 8: the ester of α-chaconine and palmitic acid (Fig. 8A), the triple oxidized α-chaconine (Fig. 8B), the ester of the β-chaconine(s) and palmitic acid (Fig. 8C), and the triple oxidized β-chaconine(s) (Fig. 8D). All four compounds were already found after 2.5 min of heating (Fig. 8E–H). During further heating, the concentration increased continuously and resulted in the highest concentrations after 60 or 120 min, respectively. In samples of similarly heated potato flesh of the same tubers, these four compounds were identified as well, but in significantly lower concentrations than in the heated peel. In Fig. 8, only compounds that are based on chaconine and esters with palmitic acid are depicted exemplarily. Similar reaction products of solanine and other fatty acids were formed as well. In fact, esters with linoleic acid could be detected in slightly higher intensity, because linoleic acid is the most prevalent fatty acid in potato lipids (Galliard, 1973). Additionally, further reaction products such as the ester of triple oxidized β-chaconine and palmitic acid and the ester of triple oxidized solanidine and palmitic acid were detected after 120 min of heating in these complex matrices as well. However, the signals of these compounds were very low, suggesting a lower concentration. In general, the formation of reaction products seems to be slower in the more complex matrix. Moreover, in the simpler models, free fatty acids were used. Obviously, in the natural matrix, lipids are esterified and embedded in different cell organelles.

Initially, it was hypothesized that the frying oil could be a source of reaction partners, as it apparently provides the dominant amount of lipid in the process of frying of potato crisps and French fries. In all experiments presented so far, no additional oil/fat was applied. Consequently, the formation of the GA fatty acid esters must originate from the endogenous fatty acids of the potato. In addition, experiments were conducted in which sunflower oil was added to the potato matrix. However, adding oil to the food-like matrix and subsequent heating did not increase the amount of reaction products that are formed substantially (Fig. S4 and Fig. S5). From a chemical point of view, this could be because the fatty acids in the frying oil are present in the form of triacylglycerols and are too nonpolar for the polar hydroxyl groups of the sugar components to react with the triacylglycerols. In contrast, this is underlined by the fact that the amphiphilic GA can be incorporated in the cell membranes by forming complexes with sterols (Milner et al., 2011). Thus, a higher reactivity of the GA with lipids of the cell membrane instead of external frying oil seems to be likely. From a food technological point of view, it is intended that only less of the frying oil enters the product during frying, as the evaporation of water and the formation of a certain crust prevent entering. A higher content of fat in potato crisps and French fries can be only expected after the water evaporated and soaking of the oil into the fine pores of the food is starting, especially when the product is cooling down (Arslan et al., 2018; Bouchon et al., 2001; Ziaiifar et al., 2008). Consequently, as α-GA are rather polar glycosides and are expected to remain in the potato product, a significant reaction with the frying oil seems to occur only at the interface of the product and the frying oil. Only limited research reported α-GA being significantly found in the frying oil (Friedman, 2006). Therefore, a significant reaction of the GA with the frying oil is not expected. However, the more non-polar the substances are becoming during α-GA conversion and modification, the higher is a certain potential for a migration into the frying oil. It is certainly necessary to conduct further, more detailed studies on the role of frying oil in the frying of actual potato products, such as potato crisps and French fries.

To evaluate toxicological and physiological aspects and the relevance of these novel reaction products regarding human diet, further investigations are needed. α-Solanine and α-chaconine can cause toxic symptoms via two mechanisms: first, disruption of (mammal) cell integrity through complexes formation with cholesterol and subsequent bulking of the membrane due to the three-dimensional structure of the sugar moiety (Keukens et al., 1995; Milner et al., 2011; Rayburn et al., 1994), and second, the inhibition of the acetyl- and buturylcholinesterase, with the steroidal alkaloid nitrogen playing a key role in this inhibition (Milner et al., 2011;Roddick, 1989; Roddick et al., 2001). Both mechanisms can hypothetically be influenced by the modified chemical structure of the reaction products formed – transformation of the sugar moiety and subsequent change of the bulking as well as the oxidation in the vicinity of the alkaloid nitrogen and subsequent change of the enzyme inhibition. In the worst case, the influence may be stronger. However, it can also be weaker. A subsequent risk assessment needs to be done in future studies. Based on the two toxicological mechanisms, this should be done by either investigations with enzyme inhibition assays or cytotoxicity experiments regarding the integrity of the cell membranes.

## 4 Conclusions

The present study provided insights into the stability of GA and their reactivity towards fatty acids in model systems as well as in a heated potato matrix. Novel GA reaction products, such as fatty acid esters and oxidized GA (Fig. 6, tentative structure suggestion), were described for the first time. These products originate from endogenous lipids and, contrary to expectations, frying oil did not seem to contribute to the formation of GA fatty acid esters. In addition to their formation in model systems, their occurrence was also demonstrated in a food-like matrix (heated potato peel powder, Fig. 8, as well as heated potato powder). Furthermore, a multitude of additional reaction products resulting from combinations of hydrolysis, oxidation, and esterification of GA were also identified in the model systems (Fig. 7). It is reasonable to assume that all these pathways may occur during the processing of real food samples such as potato crisps and French fries, too. As these reaction products have been detected in heated potato matrices, they might have the potential as markers for thermal treatment of potatoes, including the processing of widely consumed products. As GA and lipids are present at disproportionately higher levels in and near the peel of the tuber, a greater abundance of reaction products is observed in heated potato peels. Accordingly, higher levels of these reaction products are expected in crisps and French fries containing (residual) peels. Comprehensive quantification of these compounds, as well as a determination of the conditions and extent of their formation during processing, remains necessary. Subsequently, toxicological studies are required to assess their potential impact on the human diet.

However, the isolation of these compounds is essential for their comprehensive characterization, as it provides the basis for absolute structural elucidation by NMR, accurate quantification by LC–MS/MS, and subsequent risk assessment. At the same time, this step remains highly challenging due to the large number of structurally diverse compounds formed, each occurring at low concentrations. Overall, the results demonstrate that thermal processing induces multiple transformation pathways of GA, leading to the formation of previously unrecognized compounds. These findings highlight the need to extend current analytical approaches beyond native GA to include their transformation products, which may also be relevant for the toxicological evaluation of potato-based foods.

## CRediT author statement

**Keven Mittau**: conceptualization, methodology, validation, formal analysis, investigation, data curation, writing - original draft, visualization, project administration; **Christina Meyers**: conceptualization, writing - review & editing, project administration; **Harshadrai M. Rawel**: methodology, validation, resources, writing - review & editing; **Inga Smit**: writing - review & editing, project administration, funding acquisition; **Marcus Schmidt**: writing - review & editing, project administration, funding acquisition; **Sascha Rohn**: conceptualization, methodology, resources, writing - review & editing, supervision, project administration, funding acquisition.

## Declaration of generative AI and AI-assisted technologies in the writing process

During the preparation of this work, the authors used DeepL and ChatGPT in order to improve the readability and language of the manuscript. After using these tools, the authors reviewed and edited the content as needed and take full responsibility for the content of the published article.

## Declaration of competing interest

The authors declare that they have no known competing financial interests or personal relationships that could have appeared to influence the work reported in this paper.
