# Supplementary material for: Stability of potato glycoalkaloids under heating conditions – Reactions with fatty acids
Source: Curr Res Food Sci. 2026 May 15;12:101438. doi: 10.1016/j.crfs.2026.101438 (PMC13217416; doi:10.1016/j.crfs.2026.101438)
Supplement: Multimedia component 1 [file mmc1.docx]

**Stability of potato glycoalkaloids under heating conditions – Reactions with fatty acids**

Keven Mittau^1^, Christina Meyers^1,2^, Harshadrai M. Rawel^3^, Inga Smit^2^, Marcus Schmidt^4^, Sascha Rohn^1^

^1^Technische Universität Berlin, Institute of Food Technology and Food Chemistry, Kaiserin-Augusta-Allee 14, 10553 Berlin, Germany

^2^Max Rubner-Institut, Federal Research Institute of Nutrition and Food, Department of Safety and Quality of Cereals, Schützenberg 12, 32756 Detmold, Germany

^3^University of Potsdam, Institute of Nutritional Science, Arthur-Scheunert-Allee 114-116, 14558 Nuthetal, Germany

^4^Eberswalde University for Sustainable Development (HNEE), Schickler Straße 5, 16225, Eberswalde, Germany

*Corresponding author: Sascha Rohn; rohn@tu-berlin.de; +49 030 314 72583

# Supplementary Data

**Table S1A:** Fragmentation of the ester of α-chaconine and stearic acid (c.f. sections 2.5, 2.6 and 3.2; direct injection ESI(+)-HRMS^2^; CID 55 V; 29 nmol α-chaconine and 300 nmol stearic acid, heated for 5 min at 180 °C).

| **fragment ion / *m/z*** | **relative intensity** | **molecular formula based on the exact mass** | **theo. mass** | **delta (ppm)** | **proposed composition** |
| --- | --- | --- | --- | --- | --- |
| 380.3316 | 6.1% | C₂₇H₄₂N^+^ | 380.3312 | +1,11 | solanidine – H₂O |
| 381.3349 | 1.0% | C₂₆¹³CH₄₂N^+^ | 381.3345 | +0,97 | solanidine – H₂O (¹³C) |
| 398.3420 | 11.2% | C₂₇H₄₄ON^+^ | 398.3417 | +0,65 | solanidine |
| 399.3453 | 1.9% | C₂₆¹³CH₄₄ON^+^ | 399.3451 | +0,51 | solanidine (¹³C) |
| 426.3369 | 3.2% | C₂₈H₄₄O₂N^+^ | 426.3367 | +0,57 | solanidine + CO |
| 560.3949 | 2.3% | C₃₃H₅₄O₆N^+^ | 560.3946 | +0,60 | γ-chaconine |
| 586.4107 | 2.0% | C₃₅H₅₆O₆N^+^ | 586.4102 | +0,83 | γ-chaconine + C₂H₂ |
| 588.3900 | 2.8% | C₃₄H₅₄O₇N^+^ | 588.3895 | +0,88 | γ-chaconine + CO |
| 672.4476 | 2.3% | C₃₉H₆₂O₈N^+^ | 672.4470 | +0,90 | β-chaconine – H₂O – [O] |
| 688.4426 | 5.4% | C₃₉H₆₂O₉N^+^ | 688.4419 | +1,00 | β-chaconine – H₂O |
| 689.4459 | 1.7% | C₃₈¹³CH₆₂O₉N^+^ | 689.4453 | +0,92 | β-chaconine – H₂O (¹³C) |
| 706.4531 | 39.1% | C₃₉H₆₄O₁₀N^+^ | 706.4525 | +0,89 | β-chaconine |
| 707.4565 | 10.8% | C₃₈¹³CH₆₄O₁₀N^+^ | 707.4558 | +0,95 | β-chaconine (¹³C) |
| 734.4481 | 60.2% | C₄₀H₆₄O₁₁N^+^ | 734.4474 | +0,97 | β-chaconine + CO |
| 735.4515 | 15.0% | C₃₉¹³CH₆₄O₁₁N^+^ | 735.4507 | +1,03 | β-chaconine + CO (¹³C) |
| 748.4638 | 14.6% | C₄₁H₆₆O₁₁N^+^ | 748.4630 | +1,02 | β-chaconine + C₂H₂O |
| 749.4671 | 3.7% | C₄₀¹³CH₆₆O₁₁N^+^ | 749.4664 | +0,94 | β-chaconine + C₂H₂O (¹³C) |
| 764.4587 | 6.6% | C₄₁H₆₆O₁₂N^+^ | 764.4580 | +0,98 | β-chaconine + C₂H₂O₂ |
| 765.4621 | 1.7% | C₄₀¹³CH₆₆O₁₂N^+^ | 765.4613 | +1,03 | β-chaconine + C₂H₂O₂ (¹³C) |
| 834.5006 | 44.6% | C₄₅H₇₂O₁₃N^+^ | 834.4998 | +0,94 | α-chaconine – H₂O |
| 835.5040 | 13.1% | C₄₄¹³CH₇₂O₁₃N^+^ | 835.5032 | +0,99 | α-chaconine – H₂O (¹³C) |
| 972.7145 | 95.2% | C₅₇H₉₈O₁₁N^+^ | 972.7134 | +1,09 | β-chaconine + stearic acid |
| 973.7179 | 51.4% | C₅₆¹³CH₉₈O₁₁N | 973.7168 | +1,14 | β-chaconine + stearic acid (¹³C) |
| 1117.7606 | 4.5% | unknown | - | - | - |
| 1118.7726 | 100% | C₆₃H₁₀₈O₁₅N^+^ | 1118.7713 | +1,12 | α-chaconine + stearic acid |
| 1119.7761 | 9.4% | C₆₂¹³CH₁₀₈O₁₅N^+^ | 1119.7747 | +1,25 | α-chaconine + stearic acid (¹³C) |

**Table S1B:** Fragmentation of the oxidized α-solanine (c.f. sections 2.5, 2.6 and 3.2; direct injection ESI(+)-HRMS^2^; CID 55 V; 29 nmol α-solanine and 300 nmol stearic acid, heated for 5 min at 180 °C).

| **fragment ion / *m/z*** | **relative intensity** | **molecular formula based on the exact mass** | **theo. mass** | **delta (ppm)** | **proposed composition** |
| --- | --- | --- | --- | --- | --- |
| 374.2845 | 2.8% | C₂₇H₃₆N^+^ | 374.2842 | +0,62 | solanidine – H₂O – 3× H₂ |
| 392.2949 | 35.0% | C₂₇H₃₈ON^+^ | 392.2948 | +0,29 | solanidine – H₂O |
| 554.3476 | 1.5% | C₃₃H₄₈O₆N^+^ | 554.3476 | +0,04 | γ-solanine – 3× H₂ |
| 700.4057 | 76.5% | C₃₉H₅₈O₁₀N^+^ | 700.4055 | +0,27 | β₁-solanine – 3× H₂ |
| 701.4091 | 1.0 % | C₃₈¹³CH₅₈O₁₀N^+^ | 701.4089 | +0,32 | β₁-solanine – 3× H₂ (¹³C) |
| 716.4006 | 71.0% | C₃₉H₅₈O₁₁N^+^ | 716.4004 | +0,22 | β₂-solanine – 3× H₂ |
| 717.4039 | 1.1% | C₃₈¹³CH₅₈O₁₁N^+^ | 717.4038 | +0,14 | β₂-solanine – 3× H₂ (¹³C) |
| 862.4585 | 100% | C₄₅H₆₈O₁₅N^+^ | 862.4583 | +0,17 | α-solanine – 3× H₂ |

**Table S2:** Supplementary information on the mass transitions of the MRM method (c.f. sections 2.7 and 3.3; ESI(+)-LC-MS/MS method; XBridge™ BEH Amide Column (3.5 µm, 130 Å, 250 × 4.6 mm, Waters Corp., Milford, MA, USA); column temperature, 30 °C; flow rate, 0.8 mL/min; eluent A, 0.1% formic acid in water (v/v); eluent B, acetonitrile; 0 min, 10% A; 2 min, 10% A; 12 min, 50% A; 18 min, 50% A; 20 min, 10% A, 5 min post run; injection volume, 2 µL; desolvation gas, nitrogen; desolvation gas temperature, 200 °C; gas flow rate, 11 L/min; nebulizer pressure, 35 psi; sheath gas temperature, 275 °C; sheath gas flow, 11 L/min; fragmentor voltage,150 V)

| **substance** | **precursor ion / *m/z*** | **fragment ion /**  ***m/z* (quantifier)** | **voltage CID** | **other fragment ions /**  ***m/z* (qualifier)** | **retention time** |
| --- | --- | --- | --- | --- | --- |
| α-tomatine | 1034.5 | 84.1 | 95 V | 416.4 | 12.2 min |
| α-solanine | 868.5 | 98.1 | 95 V | 126.1, 398.3, 560.4, 706.5, 722.5 | 12.1 min |
| α-chaconine | 852.5 | 98.1 | 110 V | 126.1, 398.3, 560.4, 706.5 | 11.4 min |
| β_1_-solanine | 722.5 | 98.1 | 90 V | 126.1, 398.3, 560.4 | 11.3 min |
| β-GA (β_1_-chaconine, β_2_-chaconine, β_1_-solanine) | 706.5 | 98.1 | 75 V | 126.1, 398.3, 560.4 | 10.6 – 10.8 min |
| γ-GA (γ-chaconine, γ-solanine) | 560.4 | 98.1 | 75 V | 126.1, 398.3, 560.4 | 10.1 – 10.2 min |
| solanidine | 398.3 | 98.1 | 50 V | 126.1, 380.3 | 8.0 min |
| oxidized α-solanine | 862.5 | 120.1 | 170 V | 158.1, 392.3, 554.4, 700.5, 716.5 | 12.3 min |
| oxidized α-chaconine | 846.5 | 120.1 | 170 V | 158.1, 392.3, 554.4, 700.5 | 11.6 min |
| oxidized β_1_-solanine | 716.5 | 120.1 | 130 V | 158.1, 392.3, 554.4 | 11.6 min |
| oxidized β-GA | 700.5 | 120.1 | 130 V | 158.1, 392.3, 554.4 | 10.8 – 11.0 min |
| oxidized γ-GA | 554.4 | 120.1 | 90 V | 158.1, 392.3 | 10.2 – 10.3 min |
| oxidized solanidine | 392.3 | 120.1 | 150 V | 158.1, 374.3 | 8.0 min |
| α-solanine + palmitic acid – H_2_O | 1106.5 | 98.1 | 170 V | 398.3 | 8.8 – 10.3 min |
| α-chaconine + palmitic acid – H_2_O | 1090.5 | 98.1 | 170 V | 398.3 | 8.8 – 9.7 min |
| β_1_-solanine + palmitic acid – H_2_O | 960.5 | 98.1 | 150 V | 398.3 | 8.8 – 10.1 min |
| β-GA + palmitic acid – H_2_O | 944.5 | 98.1 | 150 V | 398.3 | 8.0 – 9.3 min |
| γ-GA + palmitic acid – H_2_O | 798.4 | 98.1 | 120 V | 398.3 | 8.1 – 8.5 min |
| solanidine + palmitic acid – H_2_O | 636.5 | 98.1 | 100 V | 398.3 | 5.8 min |
| α-solanine + linoleic acid – H_2_O | 1130.5 | 98.1 | 170 V | 398.3 | 8.8 – 10.3 min |
| α-chaconine + linoleic acid – H_2_O | 1114.5 | 98.1 | 170 V | 398.3 | 8.8 – 9.7 min |
| β_1_-solanine + linoleic acid – H_2_O | 984.5 | 98.1 | 150 V | 398.3 | 8.8 – 10.1 min |
| β-GA + linoleic acid – H_2_O | 968.5 | 98.1 | 150 V | 398.3 | 8.0 – 9.3 min |
| γ-GA + linoleic acid – H_2_O | 822.4 | 98.1 | 120 V | 398.3 | 8.1 – 8.5 min |
| solanidine + linoleic acid – H_2_O | 660.5 | 98.1 | 100 V | 398.3 | 5.8 min |
| α-solanine + acetic acid – H_2_O | 1130.5 | 98.1 | 150 V | 398.3 | 11.0 – 11.5 min |
| α-chaconine + acetic acid – H_2_O | 1114.5 | 98.1 | 150 V | 398.3 | 10.3 – 10.8 min |
| β_1_-solanine + acetic acid – H_2_O | 984.5 | 98.1 | 150 V | 398.3 | 10.3 – 11.2 min |
| β-GA + acetic acid – H_2_O | 968.5 | 98.1 | 150 V | 398.3 | 9.5 – 10.1 min |
| γ-GA + acetic acid – H_2_O | 822.4 | 98.1 | 120 V | 398.3 | ND |
| solanidine + acetic acid – H_2_O | 660.5 | 98.1 | 100 V | 398.3 | ND |
| ox. α-solanine + palmitic acid – H_2_O | 1100.5 | 120.1 | 170 V | 392.3 | 8.8 – 10.3 min |
| ox. α-chaconine + palmitic acid – H_2_O | 1084.5 | 120.1 | 150 V | 392.3 | 8.8 – 9.7 min |
| ox. β_1_-solanine + palmitic acid – H_2_O | 954.5 | 120.1 | 150 V | 392.3 | ND |
| ox. β-GA + palmitic acid – H_2_O | 938.5 | 120.1 | 150 V | 392.3 | 8.0 – 9.3 min |
| ox. γ-GA + palmitic acid – H_2_O | 792.4 | 120.1 | 120 V | 392.3 | 8.1 – 8.5 min |
| ox. solanidine + palmitic acid – H_2_O | 630.5 | 120.1 | 100 V | 392.3 | 5.8 min |
| ox. α-solanine + linoleic acid – H_2_O | 1124.5 | 120.1 | 170 V | 392.3 | 8.8 – 10.3 min |
| ox. α-chaconine + linoleic acid – H_2_O | 1108.5 | 120.1 | 150 V | 392.3 | 8.8 – 9.7 min |
| ox. β_1_-solanine + linoleic acid – H_2_O | 978.5 | 120.1 | 150 V | 392.3 | ND |
| ox. β-GA + linoleic acid – H_2_O | 962.5 | 120.1 | 150 V | 392.3 | 8.0 – 9.3 min |
| ox. γ-GA + linoleic acid – H_2_O | 816.4 | 120.1 | 120 V | 392.3 | 8.1 – 8.5 min |
| ox. solanidine + linoleic acid – H_2_O | 654.5 | 120.1 | 100 V | 392.3 | 5.8 min |

**Table S3:** Table of fragment ions of **Fig. 3**. Multi-stage high-resolution mass spectra of A) solanidine (*m/z* 398.3391) and B) oxidized solanidine (*m/z* 392.2923) in a reaction mixture of α-solanine and stearic acid after 20 min of heating at 180 °C. Group I: green; group II: orange; group III: blue (c.f. sections 2.5, 2.6 and 3.2).

| **solanidine: *m/z* 398.3391** | | | | |  | **oxidized solanidine: *m/z* 392.2923** | | | | |
| --- | --- | --- | --- | --- | --- | --- | --- | --- | --- | --- |
| ***m/z*** | **relative Int.** | **composition** | **theo. mass** | **delta (ppm)** | **Group** | ***m/z*** | **relative Int.** | **composition** | **theo. mass** | **delta (ppm)** |
| 115.0535 | 3.14% | C₉H₇^+^ | 115.0542 | -6.40 | **I** | 115.0535 | 1.81% | C₉H₇^+^ | 115.0542 | -6.42 |
| 117.0691 | 3.00% | C₉H₉^+^ | 117.0699 | -6.45 | **I** | 117.0691 | 2.18% | C₉H₉^+^ | 117.0699 | -6.49 |
| 119.0848 | 3.68% | C₉H₁₁^+^ | 119.0855 | -6.34 | **I** | 119.0848 | 2.14% | C₉H₁₁^+^ | 119.0855 | -6.40 |
| 120.0809 | < 0.01% |  |  |  | **II** | 120.0800 | 21.36% | C₈H₁₀N^+^ | 120.0808 | -6.47 |
| 121.1004 | 3.54% | C₉H₁₃^+^ | 121.1012 | -6.36 | **I** | 121.1004 | 3.76% | C₉H₁₃^+^ | 121.1012 | -6.39 |
| - |  |  |  |  | **III** | 122.0956 | 43.32% | C₈H₁₂N^+^ | 122.0964 | -6.54 |
| 126.1269 | 24.66% | C₈H₁₆N^+^ | 126.1277 | -6.48 | **II** | 126.1254 | 0.02% |  |  |  |
| 128.0612 | 13.27% | C₁₀H₈•^+^ | 128.0621 | -6.43 | **I** | 128.0612 | 7.53% | C₁₀H₈•^+^ | 128.0621 | -6.49 |
| 129.0690 | 12.26% | C₁₀H₉^+^ | 129.0699 | -6.44 | **I** | 129.0690 | 8.31% | C₁₀H₉^+^ | 129.0699 | -6.43 |
| 131.0847 | 15.32% | C₁₀H₁₁^+^ | 131.0855 | -6.43 | **I** | 131.0847 | 9.39% | C₁₀H₁₁^+^ | 131.0855 | -6.33 |
| 133.1003 | 14.71% | C₁₀H₁₃^+^ | 133.1012 | -6.42 | **I** | 133.1003 | 8.62% | C₁₀H₁₃^+^ | 133.1012 | -6.44 |
| 135.1160 | 2.13% | C₁₀H₁₅^+^ | 135.1168 | -6.42 | **I** | 135.1160 | 1.12% | C₁₀H₁₅^+^ | 135.1168 | -6.47 |
| 141.0690 | 14.09% | C₁₁H₉^+^ | 141.0699 | -6.28 | **I** | 141.0690 | 8.94% | C₁₁H₉^+^ | 141.0699 | -6.31 |
| 142.0768 | 19.55% | C₁₁H₁₀•^+^ | 142.0777 | -6.33 | **I** | 142.0768 | 18.91% | C₁₁H₁₀•^+^ | 142.0777 | -6.34 |
| 143.0846 | 19.60% | C₁₁H₁₁^+^ | 143.0855 | -6.32 | **I** | 143.0846 | 13.59% | C₁₁H₁₁^+^ | 143.0855 | -6.31 |
| 144.0924 | 1.38% | C₁₁H₁₂•^+^ | 144.0934 | -6.38 | **I** | 144.0925 | 0.72% | C₁₁H₁₂•^+^ | 144.0934 | -6.08 |
| 145.1003 | 28.94% | C₁₁H₁₃^+^ | 145.1012 | -6.25 | **I** | 145.1003 | 20.07% | C₁₁H₁₃^+^ | 145.1012 | -6.35 |
| 147.1159 | 24.52% | C₁₁H₁₅^+^ | 147.1168 | -6.14 | **I** | 147.1159 | 20.40% | C₁₁H₁₅^+^ | 147.1168 | -6.22 |
| 150.1268 | 12.46% | C₁₀H₁₆N^+^ | 150.1277 | -6.00 | **III** | 150.1268 | 0.33% | C₁₀H₁₆N^+^ | 150.1277 | -6.31 |
| 152.1425 | 1.14% | C₁₀H₁₈N^+^ | 152.1434 | -5.86 | **III** | 152.1423 | 0.05% | C₁₀H₁₈N^+^ | 152.1434 | -6.75 |
| 153.0690 | 2.23% | C₁₂H₉^+^ | 153.0699 | -5.69 | **I** | 153.0689 | 0.19% | C₁₂H₉^+^ | 153.0699 | -6.07 |
| 155.0846 | 6.92% | C₁₂H₁₁^+^ | 155.0855 | -5.70 | **I** | 155.0846 | 7.43% | C₁₂H₁₁^+^ | 155.0855 | -5.73 |
| 156.0925 | 1.24% | C₁₂H₁₂^+^ | 156.0934 | -5.72 | **I** | - |  |  |  |  |
| 157.1003 | 90.58% | C₁₂H₁₃^+^ | 157.1012 | -5.83 | **I** | 157.1003 | 88.03% | C₁₂H₁₃^+^ | 157.1012 | -5.81 |
| 158.0954 | < 0.01% |  |  |  | **III** | 158.0955 | 2.02% | C₁₁H₁₂N^+^ | 158.0964 | -5.94 |
| 159.1159 | 50.18% | C₁₂H₁₅^+^ | 159.1168 | -6.00 | **I** | 159.1159 | 40.68% | C₁₂H₁₅^+^ | 159.1168 | -5.96 |
| 161.1315 | 1.97% | C₁₂H₁₇^+^ | 161.1325 | -6.21 | **I** | 161.1315 | 0.86% | C₁₂H₁₇^+^ | 161.1325 | -6.35 |
| 167.0845 | 1.18% | C₁₃H₁₁^+^ | 167.0855 | -6.30 | **I** | 167.0845 | 0.35% | C₁₃H₁₁^+^ | 167.0855 | -6.12 |
| 168.0923 | 1.11% | C₁₃H₁₂•^+^ | 168.0934 | -6.04 | **I** | 168.0923 | 0.34% | C₁₃H₁₂•^+^ | 168.0934 | -6.19 |
| 169.1001 | 11.24% | C₁₃H₁₃^+^ | 169.1012 | -6.25 | **I** | 169.1001 | 6.40% | C₁₃H₁₃^+^ | 169.1012 | -6.23 |
| 171.1157 | 19.70% | C₁₃H₁₅^+^ | 171.1168 | -6.34 | **I** | 171.1158 | 16.86% | C₁₃H₁₅^+^ | 171.1168 | -6.24 |
| 173.1314 | 7.27% | C₁₃H₁₇^+^ | 173.1325 | -6.24 | **I** | 173.1314 | 5.18% | C₁₃H₁₇^+^ | 173.1325 | -6.28 |
| 175.1107 | 6.57% | C₁₂H₁₅O^+^ | 175.1117 | -6.22 | **I*** | 175.1106 | 5.43% | C₁₂H₁₅O^+^ | 175.1117 | -6.24 |
| 178.1579 | 1.11% | C₁₂H₂₀N^+^ | 178.1590 | -6.06 | **III** | - |  |  |  |  |
| 181.1001 | 1.43% | C₁₄H₁₃^+^ | 181.1012 | -6.02 | **I** | 181.1002 | 0.13% | C₁₄H₁₃^+^ | 181.1012 | -5.52 |
| 182.1079 | 1.31% | C₁₄H₁₄•^+^ | 182.1090 | -9.14 | **I** | 182.1079 | 0.67% | C₁₄H₁₄•^+^ | 182.1090 | -6.01 |
| 183.1157 | 15.82% | C₁₄H₁₅^+^ | 183.1168 | -6.33 | **I** | 183.1157 | 11.16% | C₁₄H₁₅^+^ | 183.1168 | -6.25 |
| 185.1313 | 11.40% | C₁₄H₁₇^+^ | 185.1325 | -6.36 | **I** | 185.1313 | 6.91% | C₁₄H₁₇^+^ | 185.1325 | -6.30 |
| 187.1470 | 1.75% | C₁₄H₁₉^+^ | 187.1481 | -6.24 | **I** | 187.1469 | 0.91% | C₁₄H₁₉^+^ | 187.1481 | -6.35 |
| 195.1156 | 1.83% | C₁₅H₁₅^+^ | 195.1168 | -6.19 | **I** | 195.1155 | 0.19% | C₁₅H₁₅^+^ | 195.1168 | -6.60 |
| 196.1234 | 1.38% | C₁₅H₁₆•^+^ | 196.1247 | -6.20 | **I** | 196.1234 | 0.70% | C₁₅H₁₆•^+^ | 196.1247 | -6.20 |
| 197.1312 | 28.01% | C₁₅H₁₇^+^ | 197.1325 | -6.31 | **I** | 197.1312 | 27.41% | C₁₅H₁₇^+^ | 197.1325 | -6.30 |
| 199.1469 | 4.95% | C₁₅H₁₉^+^ | 199.1481 | -6.25 | **I** | 199.1469 | 1.72% | C₁₅H₁₉^+^ | 199.1481 | -6.19 |
| 204.1734 | 6.47% | C₁₄H₂₂N^+^ | 204.1747 | -6.25 | **III** | 204.1734 | 1.67% | C₁₄H₂₂N^+^ | 204.1747 | -6.39 |
| 206.1890 | 3.59% | C₁₄H₂₄N^+^ | 206.1903 | -6.26 | **III** | 206.1750 | 0.02% |  |  |  |
| 209.1312 | 3.80% | C₁₆H₁₇^+^ | 209.1325 | -6.21 | **I** | 209.1312 | 0.97% | C₁₆H₁₇^+^ | 209.1325 | -6.33 |
| 211.1468 | 27.14% | C₁₆H₁₉^+^ | 211.1481 | -6.05 | **I** | 211.1468 | 23% | C₁₆H₁₉^+^ | 211.1481 | -6.11 |
| 213.1625 | 4.85% | C₁₆H₂₁^+^ | 213.1638 | -6.13 | **I** | 213.1625 | 2.36% | C₁₆H₂₁^+^ | 213.1638 | -6.12 |
| 218.1890 | 0.42% | C₁₅H₂₄N^+^ | 218.1903 | -6.16 | **III** | 218.1891 | 1.32% | C₁₅H₂₄N^+^ | 218.1903 | -5.78 |
| 223.1468 | 2.38% | C₁₇H₁₉^+^ | 223.1481 | -5.95 | **I** | 223.1468 | 0.64% | C₁₇H₁₉^+^ | 223.1481 | -5.89 |
| 224.1546 | 5.81% | C₁₇H₂₀•^+^ | 224.1560 | -8.46 | **I** | 224.1546 | 4.03% | C₁₇H₂₀•^+^ | 224.1560 | -5.86 |
| 225.1625 | 12.87% | C₁₇H₂₁^+^ | 225.1638 | -5.88 | **I** | 225.1625 | 10.80% | C₁₇H₂₁^+^ | 225.1638 | -5.89 |
| 227.1780 | 0.97% | C₁₇H₂₃^+^ | 227.1794 | -6.32 | **I** | 227.1780 | 0.35% | C₁₇H₂₃^+^ | 227.1794 | -6.26 |
| 237.1624 | 1.11% | C₁₈H₂₁^+^ | 237.1638 | -6.01 | **I** | - |  |  |  |  |
| 238.1701 | 2.50% | C₁₈H₂₂•^+^ | 238.1716 | -6.13 | **I** | 238.1702 | 1.53% | C₁₈H₂₂•^+^ | 238.1716 | -6.02 |
|  |  |  |  |  | **III** | 239.1655 | 0.13% | C₁₇H₂₁N•^+^ | 239.1669 | -5.74 |
| 239.1780 | 1.04% | C₁₈H₂₃^+^ | 239.1794 | -6.16 | **I** |  |  |  |  |  |
| 253.1935 | 71.88% | C₁₉H₂₅^+^ | 253.1951 | -6.36 | **I** | 253.1935 | 100% | C₁₉H₂₅^+^ | 253.1951 | -6.38 |
| 260.2357 | 2.55% | C₁₈H₃₀N^+^ | 260.2373 | -6.22 | **III** | - |  |  |  |  |
| 271.2040 | 2.65% | C₁₉H₂₇O^+^ | 271.2056 | -6.14 | **I*** | 271.2039 | 5.33% | C₁₉H₂₇O^+^ | 271.2056 | -6.27 |
| 288.2305 | 1.02% | C₁₉H₃₀ON^+^ | 288.2322 | -5.99 | **III** | - |  |  |  |  |
| 312.2666 | 1.61% | C₂₂H₃₄N^+^ | 312.2686 | -6.42 | **III** | - |  |  |  |  |
| - |  |  |  |  | **II** | 321.2431 | 7.02% | C₂₃H₃₁N•^+^ | 321.2451 | -6.36 |
| 326.2822 | 5.12% | C₂₃H₃₆N^+^ | 326.2842 | -6.22 | **III** | - |  |  |  |  |
| 327.2900 | 100.00% | C₂₃H₃₇N•^+^ | 327.2921 | -6.16 | **II** | 327.2932 | 0.03% |  |  |  |
| 339.2899 | 3.69% | C₂₄H₃₇N •^+^ | 339.2921 | -6.33 | **III** | - |  |  |  |  |
| 340.2978 | 1.58% | C₂₄H₃₈N^+^ | 340.2999 | -6.13 | **III** | - |  |  |  |  |
| 354.2770 | 1.48% | C₂₄H₃₆ON^+^ | 354.2791 | -6.14 | **III** | - |  |  |  |  |
| 354.3133 | 1.09% | C₂₅H₄₀N^+^ | 354.3155 | -6.36 | **III** | - |  |  |  |  |
| - |  |  |  |  | **II** | 362.2455 | 2.34% | C₂₅H₃₂ON^+^ | 362.2478 | -6.27 |
| 366.3133 | 1.19% | C₂₆H₄₀N^+^ | 366.3155 | -6.06 | **III** | - |  |  |  |  |
| 368.2925 | 9.49% | C₂₅H₃₈ON^+^ | 368.2948 | -6.11 | **II** | - |  |  |  |  |
| 374.2621 | < 0.01% |  |  |  | **II** | 374.2819 | 7.85% | C₂₇H₃₆N^+^ | 374.2842 | -6.22 |
| - |  |  |  |  | **II** | 376.2611 | 4.65% | C₂₆H₃₄ON^+^ | 376.2635 | -6.27 |
| - |  |  |  |  | **III** | 377.2690 | 4.51% | C₂₆H₃₅ON^+^ | 377.2713 | -7.67 |
| 380.3288 | 21.73% | C₂₇H₄₂N^+^ | 380.3312 | -6.16 | **II** | - |  |  |  |  |
| 382.3081 | 65.30% | C₂₆H₄₀ON^+^ | 382.3104 | -6.22 | **II** | - |  |  |  |  |
| 383.1856 | 3.34% | unknown |  |  | **III** | - |  |  |  |  |
| 383.3159 | 32.58% | C₂₆H₄₁ON^+^ | 383.3183 | -7.64 | **III** | - |  |  |  |  |
|  |  |  |  |  | **precursor** | **392.2923** | **20.73%** | **C₂₇H₃₈ON^+^** | **392.2948** | **-6.45** |
| **398.3391** | **168%** | **C₂₇H₄₄ON**^+^ | **398.3417** | **-6.62** | **precursor** |  |  |  |  |  |

**Figure S1:** Full mass spectrum of **Fig. 2**. High-resolution mass spectrum of potato peels after heating for 5 min at 180 °C (direct injection ESI(+)-HRMS, c.f sections 2.4, 2.6 and 3.1).

**Table S4:** Supplementary information on the full mass spectrum in figure S1. High-resolution mass spectrum of potato peels after heating for 5 min at 180 °C (direct injection ESI(+)-HRMS, c.f sections 2.4, 2.6 and 3.1).

| **fragment ion / *m/z*** | **relative intensity** | **molecular formula based on the exact mass** | **theo. mass** | **delta (ppm)** | **proposed composition** |
| --- | --- | --- | --- | --- | --- |
| 7.6% | 212.1183 | unknown | - | - | - |
| 3.3% | 215.0163 | unknown | - | - | - |
| 3.4% | 217.0889 | unknown | - | - | - |
| 1.6% | 236.0715 | unknown | - | - | - |
| 1.1% | 269.1359 | unknown | - | - | - |
| 4.1% | 297.2342 | unknown | - | - | - |
| 1.0% | 301.1411 | unknown | - | - | - |
| 2.2% | 304.2999 | unknown | - | - | - |
| 2.7% | 319.2162 | unknown | - | - | - |
| 5.1% | 326.3782 | unknown | - | - | - |
| 1.1% | 328.3211 | unknown | - | - | - |
| 1.9% | 329.2142 | unknown | - | - | - |
| 1.3% | 331.2245 | unknown | - | - | - |
| 1.1% | 332.3313 | unknown | - | - | - |
| 1.2% | 333.2402 | unknown | - | - | - |
| 2.0% | 365.1055 | unknown | - | - | - |
| 1.2% | 369.3841 | unknown | - | - | - |
| 1.0% | 378.3155 | unknown | - | - | - |
| 0.8% | 392.2945 | C₂₇H₃₈ON^+^ | 392.2948 | −0.73 | solanidine − 3× H₂ |
| 3.9% | 394.3103 | C₂₇H₄₀ON^+^ | 394.3104 | −0.39 | solanidine − 2× H₂ |
| 1.1% | 396.3259 | C₂₇H₄₂ON^+^ | 396.3261 | −0.57 | solanidine − 1× H₂ |
| 28% | 398.3415 | C₂₇H₄₄ON^+^ | 398.3417 | −0.55 | solanidine |
| 6.5% | 399.3449 | C₂₆¹³C₁H₄₄ON^+^ | 399.3451 | −0.50 | solanidine (¹³C) |
| 1.4% | 404.2582 | unknown | - | - | - |
| 2.2% | 408.2894 | unknown | - | - | - |
| 2.9% | 413.2659 | unknown | - | - | - |
| 1.8% | 414.3364 | unknown | - | - | - |
| 4.3% | 437.7495 | C₄₅H₇₄O₁₄N Na^2+^ | 437.7501 | −1.33 | α-chaconine |
| 1.5% | 438.2512 | C₄₄¹³C₁H₇₄O₁₄N Na^2+^ | 438.2518 | −1.15 | α-chaconine (¹³C) |
| 23% | 445.7468 | C₄₅H₇₄O₁₅N Na^2+^ | 445.7475 | −1.53 | α-solanine |
| 10% | 446.2485 | C₄₄¹³C₁H₇₄O₁₅N Na^2+^ | 446.2492 | −1.49 | α-solanine (¹³C) |
| 1.8% | 446.7502 | C₄₃¹³C₂H₇₄O₁₅N Na^2+^ | 446.7509 | −1.65 | α-solanine (¹³C₂) |
| 4.2% | 453.7339 | unknown | - | - | - |
| 1.3% | 453.7443 | C₄₅H₇₄O₁₆N Na^2+^ | 453.7450 | −1.63 | solasonine |
| 1.6% | 454.2355 | unknown | - | - | - |
| 1.0% | 458.7547 | C₄₇H₇₆O₁₅N Na^2+^ | 458.7554 | −1.40 | α-chaconine + acetic acid |
| 2.0% | 466.7521 | C₄₇H₇₆O₁₆N Na^2+^ | 466.7528 | −1.50 | α-solanine + acetic acid |
| 10% | 560.3941 | C₃₃H₅₄O₆N^+^ | 560.3946 | −0.88 | γ-GA |
| 3.2% | 561.3973 | C₃₂¹³C₁H₅₄O₆N^+^ | 561.3979 | −1.06 | γ-GA (¹³C) |
| 2.1% | 615.4954 | unknown | - | - | - |
| 1.9% | 630.6179 | unknown | - | - | - |
| 1.2% | 646.1478 | unknown | - | - | - |
| 1.5% | 647.6161 | unknown | - | - | - |
| 1.0% | 700.4052 | C₃₉H₅₈O₁₀N^+^ | 700,4055 | −0,35 | β-GA − 3× H₂ |
| 56% | 706.4520 | C₃₉H₆₄O₁₀N^+^ | 706.4525 | −0.69 | β-GA |
| 23% | 707.4553 | C₃₈¹³C₁H₆₄O₁₀N^+^ | 707.4558 | −0.66 | β-GA (¹³C) |
| 3.8% | 708.4586 | C₃₇¹³C₂H₆₄O₁₀N^+^ | 708.4592 | −0.72 | β-GA (¹³C) |
| 2.2% | 716.3998 | C₃₉H₅₈O₁₁N^+^ | 716.4004 | −0.74 | β_2_-solanine − 3× H₂ |
| 1.0% | 717.4032 | C₃₈¹³C₁H₅₈O₁₁N^+^ | 717.4038 | −0.82 | β_2_-solanine − 3× H₂ (¹³C) |
| 9.2% | 722.4469 | C₃₉H₆₄O₁₁N^+^ | 722.4474 | −0.68 | β_2_-solanine |
| 3.3% | 723.4503 | C₃₈¹³C₁H₆₄O₁₁N^+^ | 723.4507 | −0.66 | β_2_-solanine (¹³C) |
| 4.8% | 748.4625 | C₄₁H₆₆O₁₁N^+^ | 748.4630 | −0.75 | β-GA + acetic acid |
| 2.0% | 749.4657 | C₄₀¹³C₁H₆₆O₁₁N^+^ | 749.4664 | −0.96 | β-GA + acetic acid (¹³C) |
| 1.3% | 764.4573 | C₄₁H₆₆O₁₂N^+^ | 764.4580 | −0.82 | β_2_-solanine + acetic acid |
| 1.4% | 846.4629 | C₄₅H₆₈O₁₄N^+^ | 846.4634 | −0.64 | α-chaconine − 3× H₂ |
| 1.2% | 850.4941 | C₄₅H₇₂O₁₄N₁^+^ | 850.4947 | −0.80 | α-chaconine − H₂ |
| 0.6% | 851.4975 | C₄₄¹³C₁H₇₂O₁₄N₁^+^ | 851.4981 | −0.71 | α-chaconine − H₂ (¹³C) |
| 100% | 852.5095 | C₄₅H₇₄O₁₄N^+^ | 852.5104 | −1.03 | α-chaconine |
| 49% | 853.5129 | C₄₄¹³C₁H₇₄O₁₄N^+^ | 853.5137 | −0.93 | α-chaconine (¹³C) |
| 11% | 854.5162 | C₄₃¹³C₂H₇₄O₁₄N^+^ | 854.5171 | −1.05 | α-chaconine (¹³C₂) |
| 1.6% | 855.5187 | C₄₂¹³C₃H₇₄O₁₄N^+^ | 855.5204 | −1.88 | α-chaconine (¹³C₃) |
| 1.0% | 860.4419 | C₄₅H₆₆O₁₅N^+^ | 860.4427 | −0.95 | α-solanine − 4× H₂ |
| 3.0% | 862.4575 | C₄₅H₆₈O₁₅N^+^ | 862.4583 | −0.84 | α-solanine − 3× H₂ |
| 1.5% | 863.4609 | C₄₄¹³C₁H₆₈O₁₅N^+^ | 863.4617 | −0.81 | α-solanine − 3× H₂ (¹³C) |
| 1.3% | 866.4889 | C₄₅H₇₂O₁₅N^+^ | 866.4896 | −0.81 | α-solanine − H₂ |
| 23% | 868.5044 | C₄₅H₇₄O₁₅N^+^ | 868.5053 | −1.01 | α-solanine and solamargine |
| 10% | 869.5079 | C₄₄¹³C₁H₇₄O₁₅N^+^ | 869.5087 | −0.89 | α-solanine (¹³C) |
| 2.4% | 870.5111 | C₄₃¹³C₂H₇₄O₁₅N^+^ | 870.5120 | −1.02 | α-solanine (¹³C₂) |
| 1.8% | 884.4994 | C₄₅H₇₄O₁₆N^+^ | 884.5002 | −0.91 | solasonine |
| 0.8% | 885.5028 | C₄₄¹³C₁H₇₄O₁₆N^+^ | 885.5036 | −0.83 | solasonine (¹³C) |
| 6.4% | 894.5201 | C₄₇H₇₆O₁₅N^+^ | 894.5209 | −0.88 | a-Chac + acetic acid |
| 3.0% | 895.5236 | C₄₆¹³C₁H₇₆O₁₅N^+^ | 895.5243 | −0.76 | a-Chac + acetic acid (¹³C) |
| 2.3% | 910.5151 | C₄₇H₇₆O₁₆N^+^ | 910.5159 | −0.87 | α-solanine + acetic acid |
| 1.1% | 911.5187 | C₄₆¹³C₁H₇₆O₁₆N^+^ | 911.5192 | −0.55 | α-solanine + acetic acid (¹³C) |
| 2.0% | 944.6814 | C₅₅H₉₄O₁₁N^+^ | 944.6821 | −0.72 | β-GA + palmitic acid |
| 1.2% | 945.6847 | C₅₄¹³C₁H₉₄O₁₁N^+^ | 945.6855 | −0.77 | β-GA + palmitic acid (¹³C) |
| 1.3% | 968.6813 | C₅₇H₉₄O₁₁N^+^ | 968.6821 | −0.87 | β-GA + linoleic acid |
| 0.7% | 969.6847 | C₅₆¹³C₁H₉₄O₁₁N^+^ | 969.6855 | −0.80 | β-GA + linoleic acid (¹³C) |
| 2.0% | 1090.7389 | C₆₁H₁₀₄O₁₅N^+^ | 1090.7400 | −0.99 | α-chaconine + palmitic acid |
| 1.3% | 1091.7424 | C₆₀¹³C₁H₁₀₄O₁₅N^+^ | 1091.7434 | −0.79 | α-chaconine + palmitic acid (¹³C) |
| 0.7% | 1106.7341 | C₆₁H₁₀₄O₁₆N^+^ | 1106.7350 | −0.80 | α-solanine + palmitic acid |
| 0.3% | 1107.7378 | C₆₀¹³C₁H₁₀₄O₁₆N^+^ | 1107.7383 | −0.49 | α-solanine + palmitic acid (¹³C) |
| 1.6% | 1114.7394 | C₆₃H₁₀₄O₁₅N^+^ | 1114.7400 | −0.56 | α-chaconine + linoleic acid |
| 0.9% | 1115.7425 | C₆₂¹³C₁H₁₀₄O₁₅N^+^ | 1115.7434 | −0.81 | α-chaconine + linoleic acid (¹³C) |
| 0.4% | 1130.7344 | C₆₃H₁₀₄O₁₆N^+^ | 1130.7350 | −0.54 | α-solanine + linoleic acid |
| 0.2% | 1131.7379 | C₆₂¹³C₁H₁₀₄O₁₆N^+^ | 1131.7379 | −0.37 | α-solanine + linoleic acid (¹³C) |

**
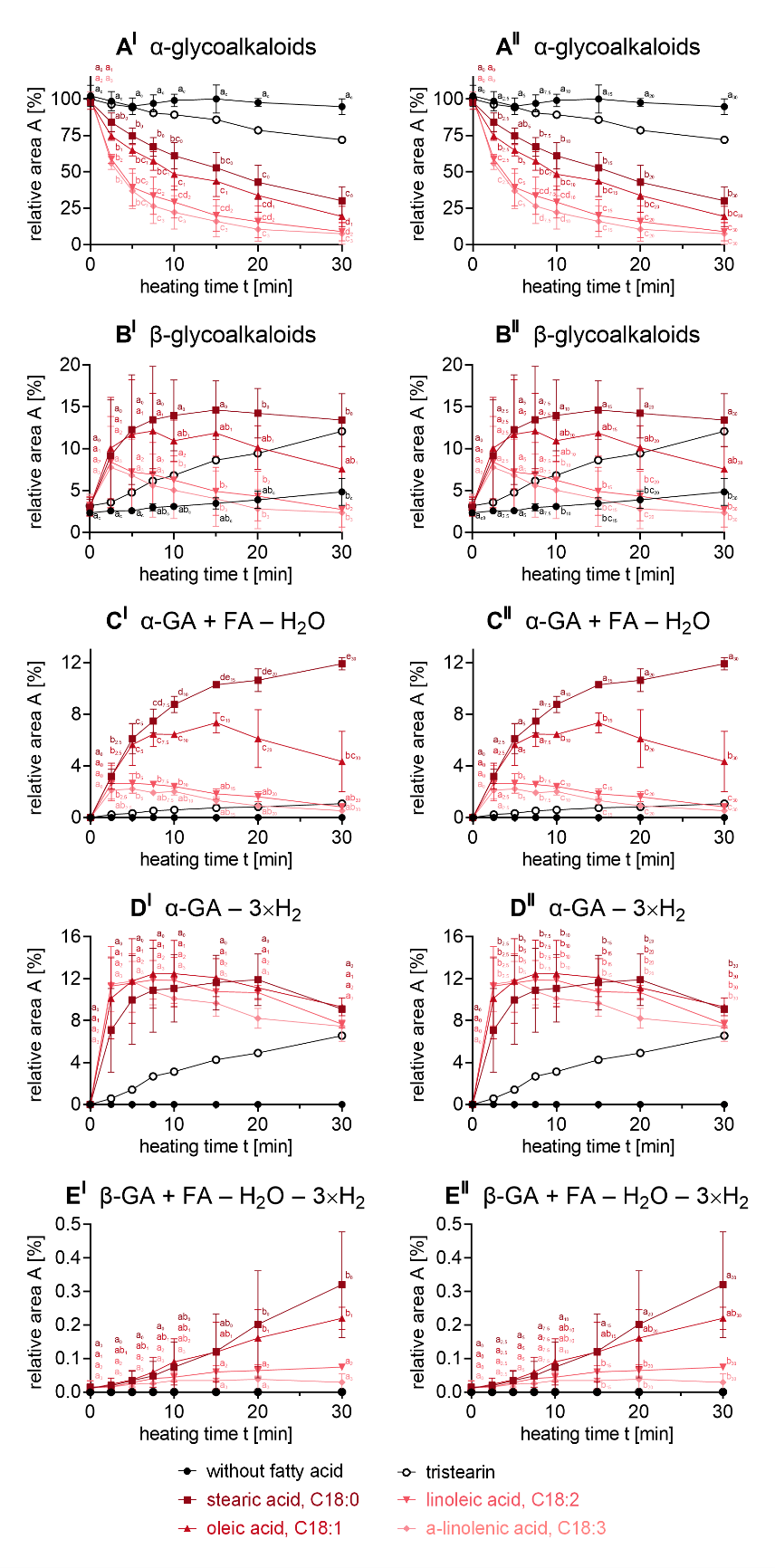
**

**Figure S2**: Addition to **Fig. 5** (c.f. section 3.4); conversion of α-GA and the formation of reaction products with fatty acids (stearic acid, oleic acid, linoleic acid, or α-linolenic acid (n = 3), tristearin (n = 1), or individual treatment ('control'; n = 3); Statistical analyses were performed by one-way ANOVA and TUKEY’s test (p < 0.05). Statistically equal values of the data points are designated by equal letters. Left side, grouped by type of fatty acid (comparison of the change during heating time); right side, grouped by heating time (comparison of the difference based on the fatty acid).


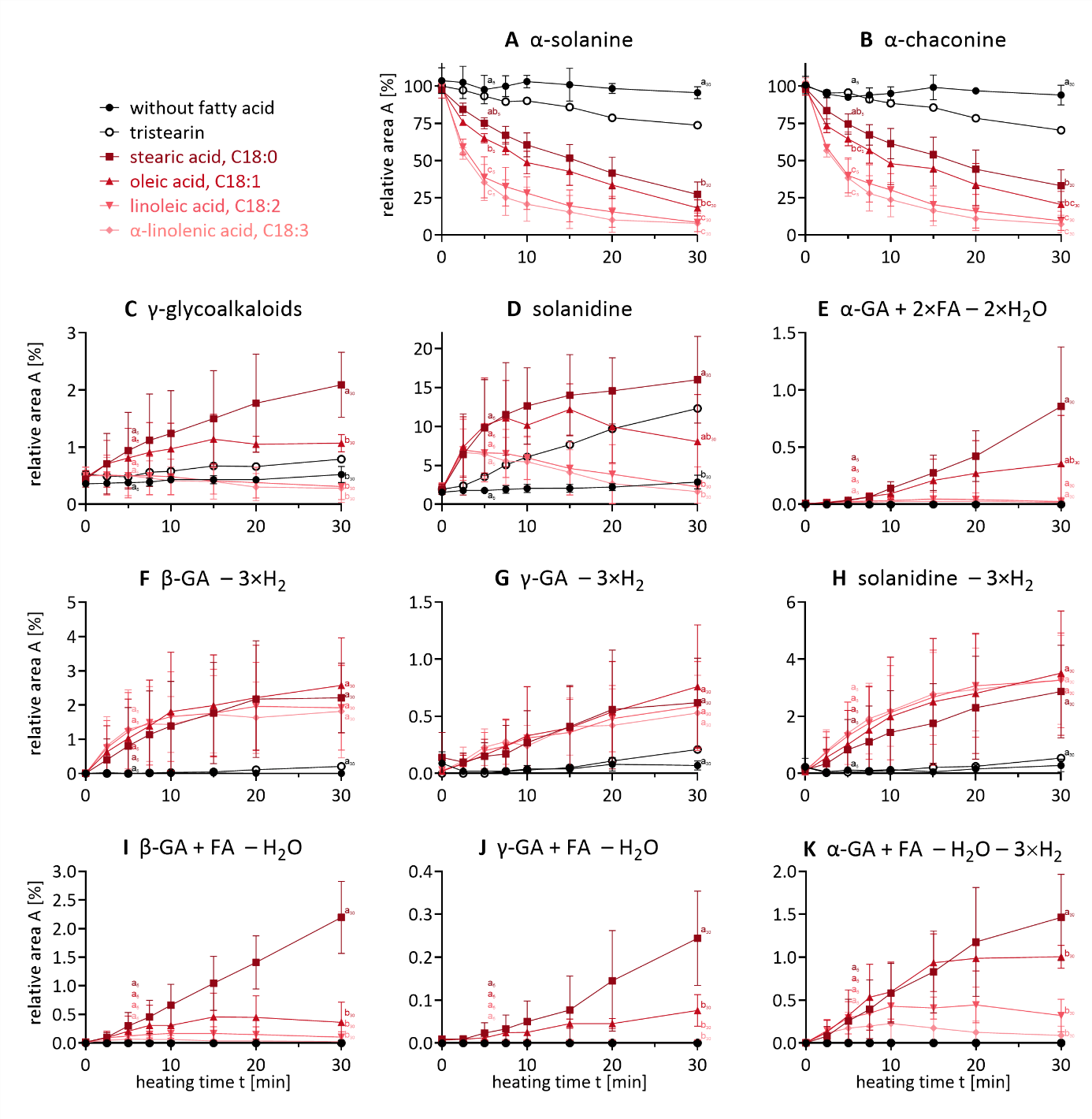


**Figure S3**: Addition to **Fig. 5** (c.f. section 3.4); further reaction products: conversion of α-chaconine and α-solanine and the formation of reaction products with fatty acids (stearic acid, oleic acid, linoleic acid, or α-linolenic acid (n = 3), tristearin (n = 1), or individual treatment ('control'; n = 3). **A)** α-solanine (*m/z* 868.5), **B)** α-chaconine (*m/z* 852.5), **C)** γ-GA (*m/z* 540.4), **D)** solanidine (*m/z* 398.3), **E)** ester of α-GA and two corresponding fatty acids (*m/z* 1372.9 and 1388.9, *m/z* 1376.9 and 1392.9, *m/z* 1380.9 and 1396.9, *m/z* 1384.9 and 1400.9), **F)** triple oxidized β-GA (*m/z* 700.5 and 716.5), **G)** triple oxidized γ-GA (*m/z* 534.4), **H)** triple oxidized solanidine (*m/z* 392.3), **I)** ester of β-GA and corresponding fatty acids (*m/z* 966.7 and 982.7, *m/z* 968.7 and 984.7, *m/z* 970.7 and 986.7, *m/z* 972.7 and 988.7), **J)** ester of γ-GA and corresponding fatty acids (*m/z* 800.6, *m/z* 802.6, *m/z* 804.6, *m/z* 806.6), **K)** ester of triple oxidized α-GA and corresponding fatty acids (*m/z* 1106.7 and 1122.7, *m/z* 1108.7 and 1124.7, *m/z* 1110.7 and 1126.7, *m/z* 1112.7 and 1128.7). Statistical analyses were performed by one-way ANOVA and TUKEY’s test (p < 0.05). Statistically equal values of the data points are designated by equal letters. Data points were grouped by heating time (comparison of the difference based on the fatty acid) and exemplarily given for 5 and 30 min.


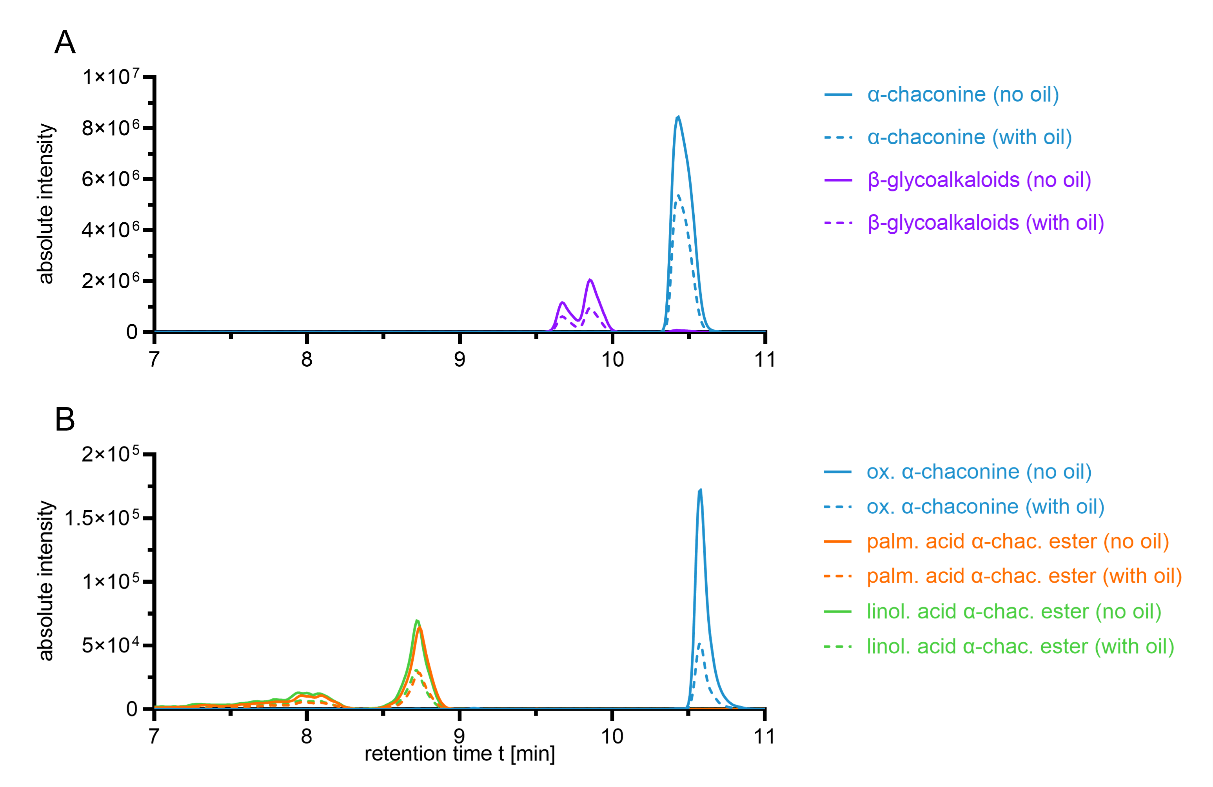


**Figure S4**: Chromatograms of selected reaction products after 5 min of heating; A) α-chaconine (m/z 852.5 ⭢ 98.1) and β-glycoalkaloids (m/z 706.5 ⭢ 98.1); B) triple oxidized α-chaconine (m/z 846.5 ⭢ 120.1), palmitic acid α-chaconine ester (m/z 1090.7 ⭢ 98.1) and linoleic acid α-chaconine ester (m/z 1114.7 ⭢ 98.1) in samples with added oil (sunflower) and without oil.


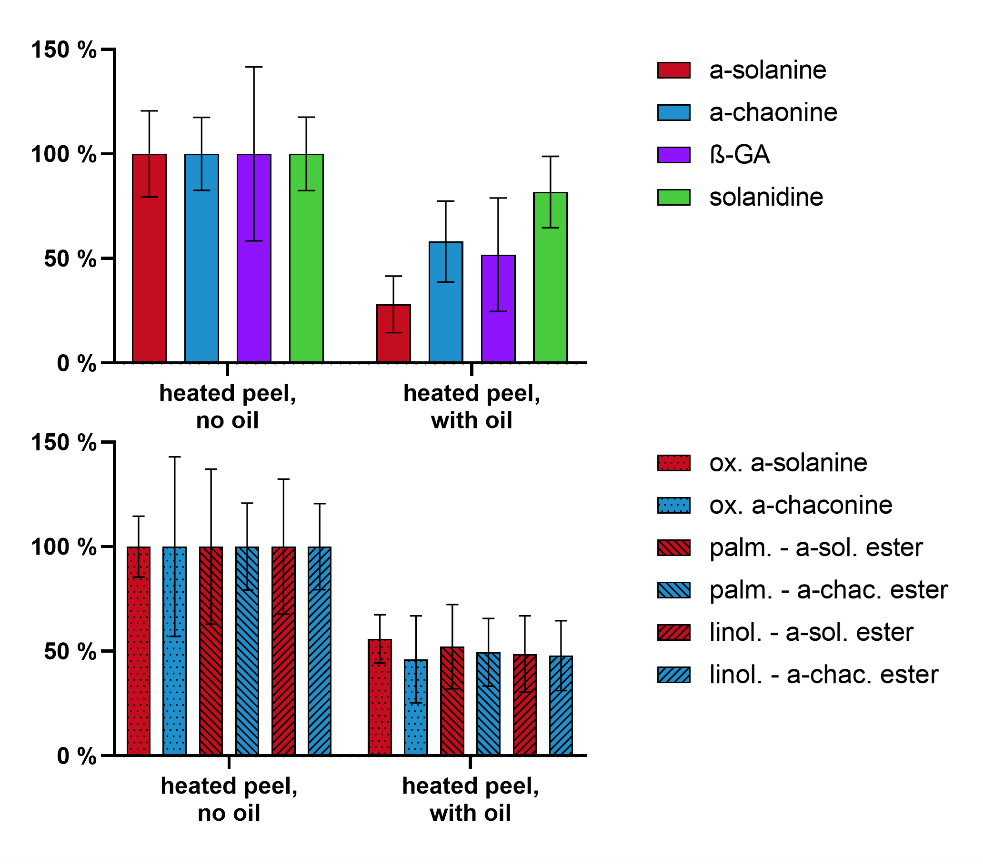


**Figure S5**: Relative concentration of α-GA and reaction products after heating of rehydrated potato peel powder with and without added oil (c.f. section 2.4). Each compound is normalized to 100% of the concentration in the samples without added oil. Samples were prepared and analyzed in triplicate by LC-MS/MS (c.f. section 2.7).

Experiments were done in triple replicates, and a representative chromatogram was chosen (Fig. S4). The oil lowers the amount of extracted GA. However, α-GA and reaction product appear to be affected to the same extent. The results suggest that the oil does not cause an increase in the formation of reaction products. Further experiments are necessary for a confirmation.

**Information about the purity of the isolated GA standard (c.f. section 2.3)**

**Figure S6**: HPLC-UV chromatogram (202 nm, c.f. section 2.8) of the isolated GA extract (300 µg/mL; c.f section 2.3) and a standard solution of α-solanine and α-chaconine (100 µg/mL each).

**Table S5:** additional data regarding the purity of the GA extract (c.f section 2.3).

|  | α-solanine | α-chaconine |
| --- | --- | --- |
| Purity of the standard  according to the manufacturer's certificate of analysis | 100 % | 98% |
| Area (standard solution) | 497 248 AU | 975 714 AU |
| Area (GA extract) | 541 949 AU | 532 136 AU |
| Content in GA standard | 36.3 % | 59.7% |
| Molecular ratio | 37.4 % | 62.6 % |

Considering various margins of error, the amount of GA (sum of α-solanine and α-chaconine) in the extract was determined as 96 ± 4% with a molar ratio of α-solanine:α-chaconine 38:62 (± 3).
